# Homodyne-based quantum random number generator at 2.9 Gbps secure against quantum side-information

**DOI:** 10.1038/s41467-020-20813-w

**Published:** 2021-01-27

**Authors:** Tobias Gehring, Cosmo Lupo, Arne Kordts, Dino Solar Nikolic, Nitin Jain, Tobias Rydberg, Thomas B. Pedersen, Stefano Pirandola, Ulrik L. Andersen

**Affiliations:** 1 Center for Macroscopic Quantum States (bigQ), Department of Physics, Technical University of Denmark, Fysikvej, 2800 Kgs. Lyngby, Denmark; 2 Department of Computer Science, University of York, York, YO10 5GH UK; 3 Department of Physics and Astronomy, University of Sheffield, Sheffield, UK; 4 Cryptomathic A/S, Åboulevarden 22, 8000 Aarhus C, Denmark

**Keywords:** Quantum physics, Quantum information

## Abstract

Quantum random number generators promise perfectly unpredictable random numbers. A popular approach to quantum random number generation is homodyne measurements of the vacuum state, the ground state of the electro-magnetic field. Here we experimentally implement such a quantum random number generator, and derive a security proof that considers quantum side-information instead of classical side-information only. Based on the assumptions of Gaussianity and stationarity of noise processes, our security analysis furthermore includes correlations between consecutive measurement outcomes due to finite detection bandwidth, as well as analog-to-digital converter imperfections. We characterize our experimental realization by bounding measured parameters of the stochastic model determining the min-entropy of the system’s measurement outcomes, and we demonstrate a real-time generation rate of 2.9 Gbit/s. Our generator follows a trusted, device-dependent, approach. By treating side-information quantum mechanically an important restriction on adversaries is removed, which usually was reserved to semi-device-independent and device-independent schemes.

## Abstract (web summary)

Security analyses for trusted quantum random number generators usually consider only classical side-information. Here, the authors fill this gap by fully characterising the experimental apparatus of a homodyne-based QRNG, assuming that the vacuum fluctuations and noise are stationary and Gaussian.

## Introduction

Random numbers are ubiquitous in modern society^1^. They are used in numerous applications ranging from cryptography, simulations, and gambling, to fundamental tests of physics. For most of these applications, the quality of the random numbers is of utmost importance. If, for instance, cryptographic keys originating from random numbers are predictable, it will have severe consequences for the security of the internet. To ensure the security of cryptographic encryption, the random numbers used to generate the secret encryption key must be completely unpredictable, private, and their randomness must be certified.

True unpredictability and privacy of the generated numbers can be attained through a quantum measurement process: by performing a projective measurement on a pure quantum state, and ensuring that the state is not an eigenstate of the measurement projector, the outcome is unpredictable and thus true random numbers can be generated^2^. Moreover, the generated numbers can be private since a pure state cannot be correlated to any other state in the universe.

Numerous different types of quantum random number generators (QRNGs) have been devised exploiting the quantum uncertainty in photon counting measurements, phase measurements, or quadrature measurements^3–5^. One particular approach of increasing interest due to its high practicality is the optical quadrature measurements of the vacuum state by means of a simple homodyne detection^6–8^. This approach combines simplicity, cost-effectiveness, chip integrability, and high generation speed.

State-of-the art security proofs for such QRNGs assumed that the information available in the environment about the measurement outcomes, so-called side information, is of classical nature^8^. Recently, quantum side information was taken into account for a source-independent QRNG^9–12^, which however requires a more complex measurement apparatus.

Furthermore, it has been assumed in the security proof that subsequent measurement outcomes of QRNGs based on homodyning of vacuum states are uncorrelated in time. Therefore, experiments dealt with the unavoidable correlations caused by the finite bandwidth of the detection system by exploiting aliasing in the sampling procedure or by using suitable post-processing algorithms^6–8,11,13–20^. Such measures usually throttle the overall rate considerably or remove the correlations only partially.

A rigorous characterization of the system is of utmost importance as any parameter uncertainty introduces a non-zero probability for system failure, i.e., the probability that the actual device does not follow the stochastic model describing the underlying physical random number generation process. Knowing the failure probability for the system is critical to its certification. Previously this metrology-grade approach was used for phase fluctuation QRNGs^21^. This includes that imperfect analog-to-digital conversion is taken into account.

Real-time field-programmable-gate-array (FPGA) implementations of randomness extraction with Gbit/s-speed using an information theoretically secure Toeplitz randomness extractor have been demonstrated recently^12,18–20,22^. Previously reported QRNG implementations achieved only moderate speeds or did not extract random numbers in real time^6–8,11,13–17^.

Here we devise a security analysis for QRNGs based on quadrature measurements of the (trusted) vacuum state that takes quantum side information into account. Our security analysis is based on the assumptions of stationarity and Gaussianity of the involved noise processes. We include correlations of measurement outcomes in the security proof as well as the imperfections of analog-to-digital conversion. We experimentally implement the QRNG and use a conservative and rigorous approach to characterize the parameters of the stochastic model that determines the amount of randomness. To establish a conservative bound with confidence intervals on the amount of vacuum fluctuations, we devise an experimental procedure based on a measurement of the transfer function (TF) of the measuring device. Using real-time Toeplitz randomness extraction implemented in an FPGA, we achieve a rate of 2.9 Gbit/s.

## Results

### Setting the stage

**Fig. 1:**
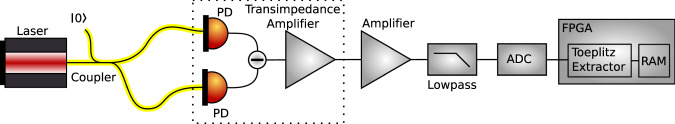
Schematic of the quantum random number generator. A 1.6 mW 1550 nm laser beam was split into two by a 3 dB fiber coupler and detected by a home-made homodyne detector based on an MAR-6 microwave amplifier from Minicircuits and two 120 μm indium–gallium–arsenide photo diodes (PD). The output of the detector was amplified with another microwave amplifier, low pass filtered at 400 MHz, and digitized with a 16 bit 1-GSample/s analog-to-digital converter (ADC). The ADC output was read by a Xilinx Kintex UltraScale field-programmable gate array (FPGA). The ADC and FPGA were hosted by a PCI Express card from 4DSP (Abaco). The FPGA was used for real-time randomness extraction based on Toeplitz hashing. Random access memory (RAM) was used to store the output.

A schematic of our QRNG is shown in Fig. 1. An arbitrary quadrature of the vacuum state is measured using a balanced homodyne detector comprising a bright reference beam, a nominal symmetric beam splitter, and two photo diodes^23^. The measurement outcomes ideally are random with a Gaussian distribution associated with the Gaussian Wigner function of the vacuum state^24^. The measured distribution, however, contains two additional independent noise sources: excess optical noise and electronic noise, thereby contributing two side channels. These must be accounted for in estimating the min-entropy of the source.

The amount of quantum randomness that can be extracted from the homodyne measurement of vacuum fluctuations is given by the leftover hash lemma against quantum side information^25,26^

1 $$\ell \ge N{H}_{\min }(X | E)-{\mathrm{log}}\,\frac{1}{2{\epsilon }_{\mathrm{hash}}^{2}}.$$

Here ${H}_{\min }(X| E)$ is the min-entropy of a single measurement outcome drawn from a random variable *X* conditioned on the quantum side information *E*, *N* is the number of aggregated samples, and *ϵ*_hash_ is the distance between a perfectly uniform random string and the string produced by a randomness extractor. It is therefore clear that we need to find the min-entropy of our practical—thus imperfect—realization in order to bound the amount of randomness. We achieve this in a two-step approach: First, we theoretically derive a bound for the min-entropy using a realistic model and express it in terms of experimentally accessible parameters. Second, we experimentally deduce these parameters through a conservative and rigorous characterization. Using such an approach, we find the worst-case min-entropy compatible with the confidence intervals of our characterization and calibration measurements, thereby obtaining a string of *ϵ*-random bits that are trustworthy with the same level of confidence.

### Theoretical analysis

The theoretical analysis of the security of the QRNG is made under the following assumptions:

- A0 The predictions of quantum mechanics are reliable.
- A1 The measurement performs homodyne detection on a single-mode and the measurement outcome is linear in the quadratures.
- A2 The quantum state that is measured is a single mode thermal state with stationary mean photon number.

The analysis of the QRNG follows a device-dependent approach, which assumes that the system (and therefore the min-entropy of the source) does not change after system characterization (A2). The quantum side information comprises all information that can be extracted from the environment of the QRNG, i.e., from the rest of the universe. Therefore, under assumptions A0–A2, the bits extracted by the QRNG are random with respect to all (quantum and classical) side channels. Following A2, homodyne detection is performed on a single optical mode in a thermal state, which at a given time is characterized by the field quadratures $\hat{q}$ and $\hat{p}$.

The physical model of our device is derived in “Methods.” There we show that our device performs the measurement

2 $$\hat{q}=g({\hat{X}}_{a}+\hat{N})\ ,$$

where *g* is a gain factor, ${\hat{X}}_{a}$ is the quadrature operator of the vacuum mode entering the central beam splitter, and $\hat{N}$ is a noise operator describing all noise sources.

In the following, we first present a theoretical analysis of a source emitting i.i.d. (independent and identically distributed) quantum states, i.e., a source of infinite bandwidth, and an ideal analog-to-digital converter (ADC). We then extend the security analysis to imperfect ADCs. Finally, we extend to a source with finite bandwidth that emits correlated (non-i.i.d.) quantum states at different times.

### Limit of identical and independent distribution

Under ideal conditions, homodyne detection would allow us to measure the quadrature of a target optical mode, which in our setting is in the vacuum state. However, as discussed in detail in “Methods,” because of experimental imperfections, this vacuum signal is mixed with noise. Therefore, the non-ideal homodyne detector measures the quadrature $\hat{q}$ of a mode, denoted in the following as *S*, that is not in the vacuum state. Following assumption A2, said state is a thermal state, which we denote as *ρ*_*S*_. We recall that a thermal state is uniquely characterized by the mean photon number *n*.

We require the random numbers to be statistically independent of any quantum or classical side information. Therefore, we need to analyze the correlations between the measured system *S* and its environment *E*. Following A0, the joint state of *S* and *E* is necessarily a pure state, ψ_*SE*_, as the combined system *SE* is by definition isolated^27^. There exist infinitely many purifications *ψ*_*SE*_ of the thermal state *ρ*_*S*_. However, these purifications are all equivalent up to local unitary transformations in the environment *E*, and thus they all have the same information content^27^. To perform our theoretical analysis, it is therefore sufficient to consider any of these purifications. We choose the two-mode squeezed vacuum (TMSV), which is a two-mode Gaussian state that purifies the thermal state^24^. The environment *E* is thus described by a single bosonic mode.

The outcome *X* of homodyne detection on a thermal state with mean photon number *n* is a continuous real-valued variable, whose probability density distribution is

3 $${p}_{X}(x)=G(x;0,{g}^{2}(1+2n))\ ,$$

where *g* is a gain factor and

4 $$G(x;\mu ,{v}^{2})=\frac{1}{\sqrt{2\pi {v}^{2}}}\ {e}^{-\frac{{(x-\mu)}^{2}}{2{v}^{2}}}$$

denotes a Gaussian in the variable *x*, with mean *μ*, and variance *v*^2^.

In our QRNG, the continuous variable *X* is mapped into a discrete and bounded variable $\bar{X}$ due to the use of an ADC with range *R* and bin size Δ*x*. We therefore consider a model in which *X* is replaced by a discrete variable $\bar{X}$ that assumes values *j* = 1, 2, …, *d* with probability mass distribution

5 $${p}_{\overline{X}}(j)=\int_{{I}_{j}}{\rm{d}}x{p}_{X}(x)\ ,$$

where *I*_*j*_s are *d* intervals that discretize the outcome of homodyne detection. This models an ideal ADC without errors.

The correlations between the discretized outcome $\bar{X}$ and the environment *E* are described by the classical-quantum (CQ) state,

6 $${\rho }_{\overline{X}E}={\mathop{\sum}\limits_{j}} \ {p}_{\overline{X}}(j)\left|j\right\rangle \left\langle j\right|\otimes {\rho }_{E}^{(j)},$$

with

7 $${\rho }_{E}^{(j)}=\frac{1}{{p}_{\overline{X}}(j)}\int_{{I}_{j}}{\rm{d}}x \ {p}_{X}(x){\rho }_{E}^{x}\ .$$

Here $\left|j\right\rangle$ are orthogonal states representing the possible discrete outcomes and ${\rho }_{E}^{x}$ describes the post-measurement quantum state of the environment. The explicit expressions of these quantities are given in “Methods,” and the full derivation is in Supplementary Note 2.

We will now quantify the rate of the QRNG in terms of the conditional min-entropy with quantum side information. Given the state ${\rho }_{\overline{X}E}$ in Eq. (6), the min-entropy of $\bar{X}$ conditioned on the environment mode reads^28^

8 $${H}_{\min }{(\bar{X}| E)}_{\rho }={{\mathop{{\mathrm{sup}}}\limits_{{\gamma }_{E}}}}\left[-{\mathrm{log}}\,\parallel {\gamma }_{E}^{-1/2}{\rho }_{\overline{X}E}\ {\gamma }_{E}^{-1/2}{\parallel }_{\infty }\right]\ ,$$

where ∥⋅∥_*∞*_ denotes the operator norm, i.e., the largest eigenvalue, and the supremum is over a density operator *γ*_*E*_ for the environment system. Here ${\gamma }_{E}^{-1/2}{\rho }_{\overline{X}E}\ {\gamma }_{E}^{-1/2}=\left({I}_{X}\otimes {\gamma }_{E}^{-1/2}\right){\rho }_{\overline{X}E}\left({I}_{X}\otimes {\gamma }_{E}^{-1/2}\right)$, where *I*_*X*_ is the identity operator on *X*. The $\mathrm{log}\,$ has base 2.

In “Methods,” we compute a lower bound on this quantity following a particular choice for *γ*_*E*_. The final result (which includes an optimization over the gain *g*—see “Methods” for the unoptimized result) is

9 $${H}_{\min }(\bar{X}| E)\ge -\mathrm{log}\,\left[{{\Gamma }}(n)\ {\rm{erf}}\left(\frac{{{\Delta }}x}{2{g}_{* }^{\prime}}\right)\right]\ ,$$

where

10 $${{\Gamma }}(n)={\left(\sqrt{n}+\sqrt{n+1}\right)}^{2}\ ,$$

and ${g}_{* }^{\prime}$ is implicitly defined by the equation

11 $${\rm{erf}}\left(\frac{{{\Delta }}x}{2{g}_{* }^{\prime}}\right)=\frac{1}{2}{\rm{erfc}}\left(\frac{R}{{g}_{* }^{\prime}}\right)\ .$$

### ADC digitization noise

The above result assumed an ADC without digitization errors and noise. However, those imperfections reduce the extractable min-entropy. Given the true digitization outcome *j*, the noise replaces it with a different, possibly random, output *f*. For any given *f*, we count up to *M* possible true values *j* that map into *f*. In “Methods,” we show that this reduces the min-entropy by at most $\mathrm{log}\,M$ bits, i.e.,

12 $${H}_{\min }(\bar{X}| E)\ge {H}_{\min }{(\bar{X}| E)}^{{\rm{ideal}}}-\mathrm{log}\,M\ ,$$

with ${H}_{\min }{(\bar{X}| E)}^{{\rm{ideal}}}$ given in Eq. (9).

### Beyond i.i.d.: stationary Gaussian process

We now consider the more realistic scenario of finite bandwidth. In the experimental implementation, the finite detection bandwidth, described by the impulse response of the detector, defines the temporal mode of the measured quantum state. Correlations arise due to the temporal overlap of the different modes. The process is still stationary and Gaussian (A2), however, not i.i.d. Here we use theoretical tools from information theory^29^ and signal processing^30^ to analyze this stationary Gaussian process. We first obtain a virtual i.i.d. model for the non-i.i.d. process. Then we apply the results of the previous section to compute a lower bound on the min-entropy with quantum side information of said virtual i.i.d. model.

The analysis deals with two stochastic processes. One is the outcome *X* of the homodyne measurement. The second stochastic process, denoted as *U*, describes the excess noise, i.e., all fluctuations in the measurement that are not purely vacuum fluctuations, including electronic noise of the detector and intensity noise of the local oscillator laser. Both *X* and *U* are stationary and Gaussian processes (A2). When a measurement is performed at a given time *t*, the homodyne outcome is denoted as *X*_*t*_. Similarly, we denote as *U*_*t*_ the excess noise at time *t*.

The homodyne measurement outcome *X*_*t*_ comprises several components. Part of it comes from pure vacuum fluctuations and part comes from the excess noise. However, because of the finite bandwidth, *X*_*t*_ also contains a component that is determined by past measurement outcomes, denoted as *X*_<*t*_. The component from past measurement outcomes is considered as side information.

We write the variance of *X*_*t*_ as ${\sigma }^{2}={\sigma }_{X}^{2}+\zeta$, where *ζ* accounts for the fluctuations of *X*_<*t*_, and ${\sigma }_{X}^{2}$ accounts for all fluctuations that are independent of the past, i.e., the variance of *X*_*t*_ conditioned on *X*_<*t*_. The conditional variance ${\sigma }_{X}^{2}$ accounts for both pure vacuum fluctuations and for the excess noise. The conditional variance of the excess noise is denoted as ${\sigma }_{U}^{2}$, and the variance of pure vacuum fluctuations is thus obtained as ${\sigma }_{X}^{2}-{\sigma }_{U}^{2}$. Below we develop a theory that allows us to determine the quantities ${\sigma }_{X}^{2}$, *ζ*, and ${\sigma }_{U}^{2}$.

Let us first consider the stochastic process *X*. Given the time series of measured values ${x}_{{t}_{k}}$, $\hat{x}(\lambda)={\sum }_{k}{x}_{{t}_{k}}{e}^{ik\lambda }$ is the Fourier transform, for *λ* ∈ [0, 2*π*]. The power spectral density (PSD) is then defined as ${f}_{X}(\lambda)=| \hat{x}(\lambda){| }^{2}$. The variance *σ*^2^ and the PSD can be both estimated experimentally. In turn, from the PSD we can estimate the entropy rate^29,30^,

13 $$h(X)=\frac{1}{2}{\mathrm{log}}\,(2\pi e{\sigma }_{X}^{2})\ ,$$

where

14 $${\sigma}_{X}^{2} = \frac{1}{2\pi e} {2}^{\int_{0}^{2\pi } \frac{d\lambda }{2\pi }{\mathrm{log}}\,[2\pi e{f}_{X}(\lambda)]}$$

is the conditional variance. The same formal relation links the PSD and the entropy rate of the excess noise *U*,

15 $$h(U)=\frac{1}{2}\mathrm{log}\,(2\pi e{\sigma }_{U}^{2})\ ,$$

where

16 $${\sigma }_{U}^{2}=\frac{1}{2\pi e}{2}^{\int_{0}^{2\pi }\frac{d\lambda }{2\pi }\mathrm{log}\,[2\pi e{f}_{U}(\lambda)]}$$

is the conditional variance of the excess noise.

Because of the finite bandwidth of the measuring apparatus, both the homodyne outcome *X*_*t*_ and excess noise *U*_*t*_, at a given time *t*, are correlated with their values at previous times. To filter out the effects of these correlations, we consider the probability density distribution of *X*_*t*_, conditioned on all past homodyne measurement outcomes,

17 $${p}_{{X}_{t}}({x}_{t}| {x}_{\,{<}\,t})=G({x}_{t};{\mu }_{t},{\sigma }_{X}^{2})\ ,$$

where *x*_*t*_ denotes the possible values of the variable *X*_*t*_ at time *t*, *x*_<*t*_ denotes the collection of values of all homodyne measurement outcomes at times $t^{\prime} \, < \, t$, and *μ*_*t*_ is the conditional mean value of *X*_*t*_. Note that, if *p*(*x*_1_, *x*_2_, …*x*_*n*_) is a multivariate Gaussian probability distribution, the conditional distribution *p*(*x*_1_∣*x*_2_, …*x*_*n*_) is also Gaussian. Also note that ${\sigma }_{X}^{2}$ does not depend on time because *X* is stationary (this follows, for example, from Eq. (13)). Although the mean value *μ*_*t*_ may depend parametrically on the past values *x*_<*t*_, the random variable *X*_*t*_ is (by definition) conditionally independent of previous homodyne outcomes. Therefore, we can formally describe it—once the previous measurement outcomes are known—as the outcome of a measurement applied on correlation-free quantum state with variance ${\sigma }_{X}^{2}$. We thus identify (using the notation of Eq. (3)):

18 $${\sigma }_{X}^{2}\equiv {g}^{2}(1+2n)\ .$$

We can then write the (unconditional) variance *σ*^2^ as

19 $${\sigma }^{2}={g}^{2}(1+2n)+\zeta \ ,$$

which allows us to obtain $\zeta ={\sigma }^{2}-{\sigma }_{X}^{2}$.

In summary, we have defined an effective i.i.d. model for the non-i.i.d. signal. The i.i.d. model is characterized by the parameters *n* and *g* in Eq. (18). To determine these parameters, we need a second equation in addition to Eq. (18). Such a second equation is obtained through the conditional variance of the excess noise.

For the excess noise *U*_*t*_, we can similarly write the probability density distribution conditioned on past values, i.e.,

20 $${p}_{{U}_{t}}({u}_{t}| {u}_{\,{<}\,t})=G({u}_{t};{\nu }_{t},{\sigma }_{U}^{2})\ ,$$

where *u*_*t*_ denotes the possible values of the variable *U*_*t*_ at time *t*, *u*_<*t*_ denotes its past values, and *ν*_*t*_ is the conditional mean value of *U*_*t*_. The quantity of interest is the conditional excess noise variance ${\sigma }_{U}^{2}$. We identify the latter with the variance of the excess noise in the i.i.d. model:

21 $${\sigma }_{U}^{2}\equiv 2{g}^{2}n\ .$$

By inverting Eqs. (18) and (21), we obtain the parameters *n* and *g* of the i.i.d. model of the non-i.i.d. process,

22 $$g=\sqrt{{\sigma }_{X}^{2}-{\sigma }_{U}^{2}}\ ,$$

23 $$n=\frac{1}{2}\frac{{\sigma }_{U}^{2}}{{\sigma }_{X}^{2}-{\sigma }_{U}^{2}}\ .$$

Finally, we need to account for the term *ζ*, which describes the fluctuations due to past measurements. We incorporate this in the variance of the excess noise and redefine

24 $$n\to n+\frac{\zeta }{2{g}^{2}}=\frac{1}{2}\frac{{\sigma }^{2}}{{\sigma }_{X}^{2}-{\sigma }_{U}^{2}}-\frac{1}{2}\ .$$

**Fig. 2:**
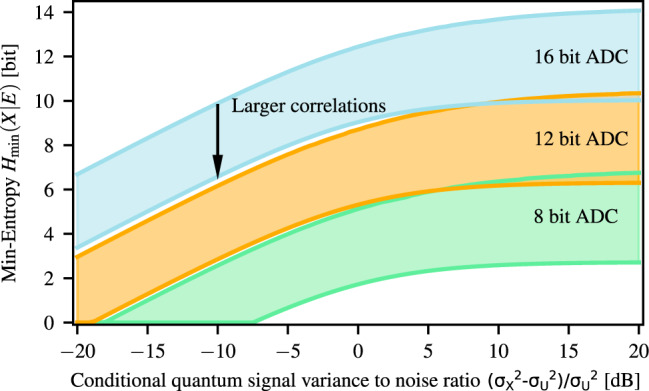
Min-entropy versus the conditional quantum signal-to-noise ratio. Min-entropy for 8-, 12-, and 16-bit analog-to-digital converter (ADC) resolution versus the ratio of conditional variance of the vacuum fluctuations and the conditional variance of the excess noise, $({\sigma }_{X}^{2}-{\sigma }_{U}^{2})/{\sigma }_{U}^{2}$. Here ${\sigma }_{X}^{2}$ and ${\sigma }_{U}^{2}$ are the conditional variance of the measurement outcomes and of the excess noise, respectively. The shaded areas indicate the regions between low correlations (${\sigma }_{X}^{2}/{\sigma }^{2}=0.99$), upper trace and high correlations (${\sigma }_{X}^{2}/{\sigma }^{2}=0.1$), lower trace. Thereby *σ*^2^ is the variance of the measurement outcomes, which has been optimized to obtain the highest min-entropy. The ADC is assumed to be ideal without digitization errors.

In conclusion, we use this virtual i.i.d. model to compute a lower bound for the min-entropy of the non-i.i.d. process, where the values for *g* and *n* in Eq. (3) are given in Eq. (22) and (24), respectively. In turn, this allows us to estimate the min-entropy rate using Eq. (9) (see also Eqs. (62) and (67) in “Methods”). This is plotted in Fig. 2 for varying excess noise, ADC resolution, and temporal correlations. The *x*-axis of the plot is the ratio of the conditional variance of the vacuum fluctuations and the excess noise, i.e., the quantum noise to excess noise ratio of the virtual i.i.d. process. If, as assumed for the plot in Fig. 2, the homodyne measurement outcomes and the excess noise have similar temporal correlations, this ratio is independent of the amount of correlations. The amount of correlations present in the system is instead characterized by the ratio ${\sigma }_{X}^{2}/{\sigma }^{2}$, which takes the value of 1 for an i.i.d. process and becomes smaller for increasing temporal correlations. For each ADC resolution, the upper traces in Fig. 2 show the extractable min-entropy when almost no correlations are present. Obviously, stronger correlations yield lower randomness.

Similar to the result for classical side information^8^, we show that random numbers can in principle be generated for noise treated as quantum side information as well and even in the large excess noise regime. This is due to the fact that relatively small vacuum fluctuations can give a substantial contribution to the entropy if the ADC resolution is sufficiently high. This property is preserved even when a large amount of temporal correlations is present in the recorded data (lower traces in Fig. 2). However, as discussed below, increasing the precision may not necessarily lead to an increase in the min-entropy in the presence of digitization errors.

### System characterization

To be able to apply the theoretical result obtained above to our experimental implementation, we need to provide evidence that our implementation indeed fulfills the assumptions. This is in fact a difficult task and a detailed discussion can be found in “Methods.”

We are now in a position to estimate the min-entropy through characterization of our set-up. According to the theoretical analysis, the min-entropy can be found by determining the variance *σ*^2^ as well as the conditional variances of the homodyne measurement outcomes ${\sigma }_{X}^{2}$ and the excess noise ${\sigma }_{U}^{2}$. To obtain a conservative, and thus reliable, estimate of the min-entropy, it is important that the measurement of these parameters does not rely on any ideality assumptions of the homodyne detector.

The first two parameters *σ*^2^ and ${\sigma }_{X}^{2}$ can be directly established from the PSD *f*_*X*_(*λ*) of the homodyne measurement outcomes. The excess noise parameter ${\sigma }_{U}^{2}$ is, however, more involved as its amount is determined by several sources whose individual contributions is too cumbersome to determine. Our goal is thus to establish the PSD of the excess noise *f*_*U*_(*λ*) by determining the contribution of the vacuum fluctuations to the total noise. ${\sigma }_{U}^{2}$ can then be computed from *f*_*U*_(*λ*) = *f*_*X*_(*λ*) − *f*_vac_(*λ*), where *f*_vac_(*λ*) is the PSD of the vacuum fluctuations.

**Fig. 3:**
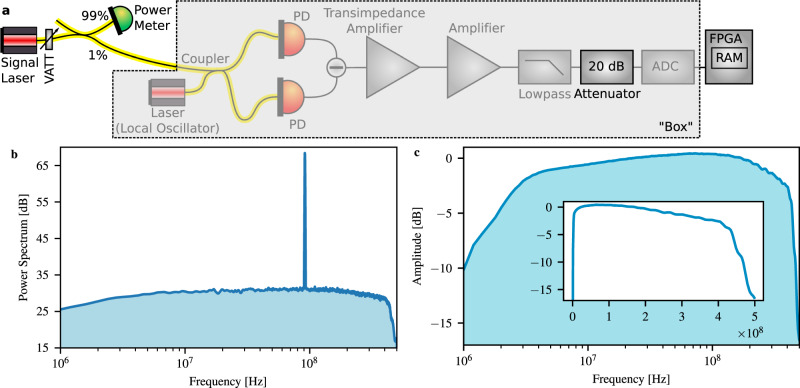
Characterization of the transfer function of the detection system to obtain the vacuum fluctuation noise level. **a** Experimental set-up for the characterization. VATT variable optical attenuator, PD photo diode, ADC analog-to-digital converter, FPGA field-programmable gate array, RAM random access memory. **b** Power spectrum from a typical measurement. The transfer function is determined by the amplitude of the beat note. **c** Transfer function of the homodyne detector and the electronics including the analog-to-digital converter. Inset: transfer function with linear frequency scale.

To establish a lower bound on *f*_vac_(*λ*), we basically consider the homodyne detector as a box (see Fig. 3a) with a quantum state input and an input–output relation given by Eq. (2) with unknown parameters. Our strategy is thus to measure the TF of the box by probing it with known quantum states and to use this result to conservatively calibrate the PSD of the vacuum fluctuations. This method allows us to establish a lower bound on the vacuum fluctuations under all experimental conditions, in particular where other noise sources couple into the detector, e.g., intensity noise of the laser due to imperfect common-mode rejection or stray light coupling into the signal port—likely to be an issue with integrated photonic chips.

The TF of the box is measured by injecting a coherent state in the form of a second laser beam (independent of the local oscillator laser) with low power *P*_sig_ into the signal port of the beam splitter as displayed in Fig. 3a. A typical beat signal is shown in Fig. 3b obtained by computing an averaged periodogram from the sampled signal. We record the TF(*ν*) by scanning the frequency of the signal laser. At each difference frequency *ν*, we determine the power of the beat signal and normalize it to *P*_sig_. At high signal-to-noise ratio, the root-mean-square power of the beat signal is purely a function of the coherent state amplitude (determined by the signal laser power). It is independent of the noise of the detector, since the second term in Eq. (2), the noise term, can be neglected. The first term depending on the quadrature operator ${\hat{X}}_{a}$ can be decomposed into a dominating term depending on the coherent state amplitude and a negligible term depending on the noise of the input state, rendering the root-mean-square power independent of the laser noise properties.

Since the vacuum noise was amplified to optimally fill the range of the ADC, we used a 20-dB electrical attenuator with flat attenuation over the frequency band of interest to avoid saturation, see Fig. 3a. The result of the TF characterization, normalized to a maximum gain of 1, is shown in Fig. 3c.

Given the linearity of the detector (A1), we obtain the PSD of the vacuum fluctuations by multiplying the TF(*ν*) with the shot noise energy $\hbar\omega_L$ contained in 1 Hz bandwidth, where $\hbar$ is Planck’s constant and *ω*_*L*_ is the angular frequency of the local oscillator laser. By modeling the inner workings of the box, we confirm in Supplementary Note 5 that with this procedure we indeed obtain a lower bound on the PSD of the vacuum fluctuations.

**Fig. 4:**
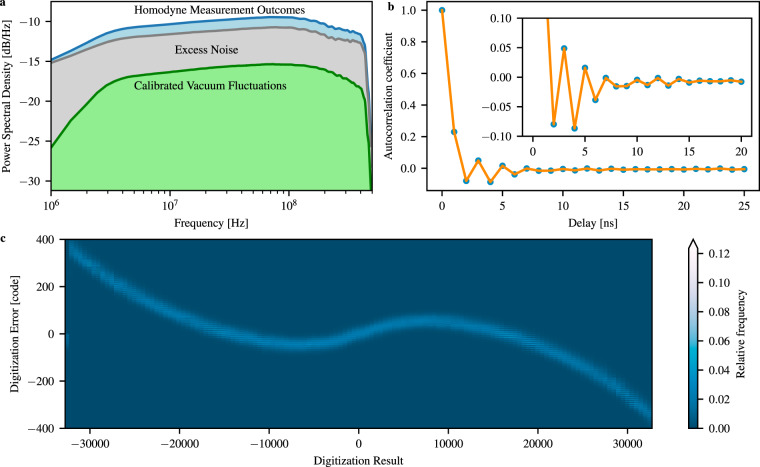
Experimental results. **a** The figure shows the power spectral densities (PSDs) of the measurement outcomes, the calibrated vacuum fluctuations (obtained by the system characterization), and the excess noise (obtained by subtracting the PSD of the vacuum fluctuations from the PSD of the measurement outcomes). **b** Autocorrelation coefficients calculated from the measured samples and averaged 1000 times. The inset shows a zoom. **c** Relative frequency of the digitization error of the analog-to-digital converter (ADC) with respect to the digitization results. The non-linearity and digitization noise of the ADC leads to a large reduction of the min-entropy.

The conservatively estimated PSD of the vacuum fluctuations is shown in Fig. 4a together with the actually measured PSD of the signal. The spectra are clearly colored which indicates that the data samples are correlated and therefore non-i.i.d. This is further corroborated in Fig. 4b, where the autocorrelation of the homodyne measurement outcomes is plotted. It justifies the importance of using the min-entropy relation associated with non-i.i.d. samples.

From the PSDs, we calculate the three parameters for obtaining the min-entropy, which are summarized in Table 1. By minimizing the min-entropy over the confidence set of the estimated parameters, we obtain 10.74 bit per 16-bit sample with a failure probability of *ϵ*_PE_ = 10^−10^ (i.e., the probability that the actual parameters are outside the confidence intervals) under the assumption of an ideal ADC.

**Table 1:** Summary of the parameters determined by system characterization.

Parameter	Value
*σ*^2^	3.96 × 10^7^ ± 0.09 × 10^7^
${\sigma }_{X}^{2}$	3.29 × 10^7^ ± 0.07 × 10^7^
${\sigma }_{U}^{2}$	2.49 × 10^7^ ± 0.06 × 10^7^
Conditional quantum to excess noise ratio	−4.9 dB
Temporal correlations ${\sigma }_{X}^{2}/{\sigma }^{2}$	0.83
Min-entropy, ideal ADC	10.74 bit
Reduction due to ADC digitization error	7.23 bit
Min-entropy	3.51 bit
Calculated secure length	1027 bit
Extracted length	1024 bit

Variance of the measurement outcomes *σ*^2^, the conditional variance of the measurement outcomes ${\sigma }_{X}^{2}$, and the conditional variance of the excess noise ${\sigma }_{U}^{2}$ with their confidence intervals for *ϵ*_PE_ = 10^−10^. The calculated min-entropy for an ideal analog-to-digital converter (ADC) minimized over the confidence intervals, the reduction due to ADC imperfections with *ϵ*_ADC_ = 2 × 10^−6^, the secure length according to the leftover hash lemma, and the length of the extracted random sequence in the experiment.

Finally, we characterized the digitization error of our ADC, which is shown in Fig. 4c. The measurement protocol is described in Supplementary Note 3. The reduction of the min-entropy due to the digitization error is 7.23 bit with a confidence of 2 × 10^−6^ as 500,000 measurements have been used to construct the histogram for each digitization result. Thus this yields a total min-entropy of 3.51 bit. This relatively large reduction is due to the fact that our ADC is four-way interleaved and has a large analog bandwidth.

## Discussion

We have demonstrated a QRNG based on the measurement of vacuum fluctuations with real-time extraction at a rate of 2.9 Gbit/s and security against quantum side information. Our QRNG has a strong security guarantee with a failure probability of ${N}^{\prime}\cdot {\epsilon }_{\text{hash}}+{\epsilon }_{\text{PE}}+{\epsilon }_{\text{ADC}}+{\epsilon }_{\text{seed}}={N}^{\prime}\cdot 1{0}^{-32}+3\times 1{0}^{-10}+2\times 1{0}^{-6}+{\epsilon }_{\text{seed}}$, where ${N}^{\prime}$ is the number of QRNG runs in the past with the same seed for the randomness extractor, *ϵ*_hash_ is the security parameter related to the removal of side information [see Eq. (1)], *ϵ*_PE_ = 10^−10^ is the security parameter of the estimation of one parameter, *ϵ*_ADC_ = 2 × 10^−6^ is related to the confidence of the digitization error measurement, and *ϵ*_seed_ describes the security of the random bits used for seeding the randomness extractor. Since quantum side information from the past has to be taken into account, *ϵ*_hash_ grows with time^2^.

We chose *ϵ*_hash_ = 10^−32^ to keep ${N}^{\prime}{\epsilon }_{\text{hash}}$ low enough to, in principle, be able to generate Gaussian random numbers with security *ϵ* = 10^−9^ for a single execution of a continuous variable quantum key distribution (QKD)^5^ protocol with 10^10^ transmitted quantum states even after 10 years of continuous operation of the QRNG. See Supplementary Note 6 for details. We note, however, that in our case the *ϵ*-security parameter is limited by *ϵ*_ADC_. In our experiment, the seed bits were chosen with a pseudo-random number generator, which does not allow us to give a security guarantee for *ϵ*_seed_. The generated random numbers passed both the Dieharder^31^ and the NIST 800-90B^32^ statistical batteries of randomness tests.

Due to the choice of a very small *ϵ*_hash_, the real-time speed of our QRNG was limited to 2.9 Gbit/s by the input size of the Toeplitz extractor required by our FPGA implementation. Without limitations to the matrix size, a speed of 3.5 Gbit/s could be reached. The main limitation to the available min-entropy is the ADC digitization error.

Our QRNG is suited for use in high-speed QKD links, for instance, in GHz clocked discrete variable^33^ as well as in high-speed continuous-variable QKD (CVQKD)^34^. For Gaussian-modulated CVQKD, the uniform random number distribution has to be converted to a Gaussian distribution, which requires a larger random number generation rate. Furthermore, QKD requires composable security and a guarantee of privacy of the random numbers as provided by our system.

Further developments to guarantee reliable operation over a long time and to fulfill requirements by certification authorities would need to include power-on self-tests and online testing of the parameters in the security analysis as well as the generated random numbers. Finally, the removal of the Gaussianity and stationarity assumptions in the security analysis, which are in practise difficult to verify, would further strengthen the security of the QRNG.

## Methods

### Physical model

**Fig. 5:**
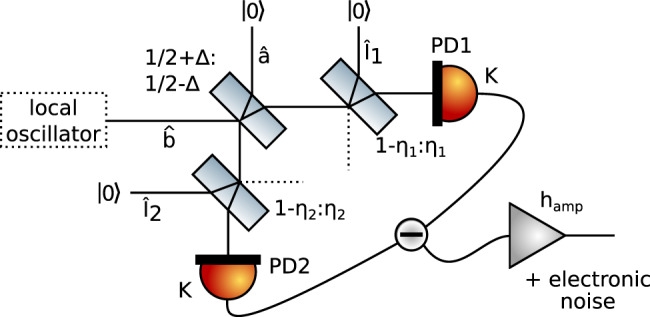
Physical model of the QRNG showing the involved modes. The local oscillator described by the mode $\hat{b}$ interferes with the vacuum state described by mode $\hat{a}$ at a beam splitter with reflectivity $\frac{1}{2}+{{\Delta }}$. The photo diode efficiencies are modeled as beam splitters with transmittivities *η*_1_ and *η*_2_, where the output modes from the central beam splitter are interfered with vacuum modes ${\hat{l}}_{1}$ and ${\hat{l}}_{2}$. The difference of the two photo currents from photo diodes PD1 and PD2, each generated by the light described by conversion factor *K*, is amplified electronically by *h*_amp_, during which electronic noise is added to the output of the detector.

Here we will develop a physical model of the QRNG using a description of optical modes by annihilation and creation operators in the Heisenberg picture^35^. A schematic of our detector depicting the involved modes and parameters is shown in Fig. 5. Mode operators $\hat{a}$ and $\hat{b}$ denote the signal and local oscillator, respectively. The signal and the local oscillator are mixed at the central beam splitter, which, under ideal conditions, has 50% splitting ratio. In our model, we consider that the splitting ratio of the central beam splitter may deviate from perfect balancing by Δ. The optical modes at the output of the central beam splitter are measured by a pair of photo diodes, with quantum efficiencies *η*_1_ and *η*_2_, respectively. The non-unit efficiencies are modeled by introducing the auxiliary modes ${\hat{l}}_{1}$ and ${\hat{l}}_{2}$. Opto-electrical conversion is described by the constant *K*.

The local oscillator laser mode $\hat{b}$ can be written as $\hat{b}=\langle \hat{b}\rangle +\delta \hat{b}\equiv \beta +\delta \hat{b}$, where $\langle \hat{b}\rangle$ is the expectation value and $\delta \hat{b}$ describes the fluctuations. We operate our homodyne detector in the strong local oscillator regime, so that products of operators describing fluctuations are negligible: $\delta \hat{x}\delta \hat{y}\approx 0$. We note that with local oscillator photon flux in the range of 10^15^ the detector operates deep within the strong local oscillator regime.

The modes that are detected by photo detection are given by

25 $$\hat{c}=\sqrt{{\eta }_{1}}\left(\sqrt{\frac{1}{2}-{{\Delta }}}\ \hat{b}+\sqrt{\frac{1}{2}+{{\Delta }}}\ \hat{a}\right)+\sqrt{1-{\eta }_{1}}\ {\hat{l}}_{1}\,,$$

26 $$\hat{d}=\sqrt{{\eta }_{2}}\left(\sqrt{\frac{1}{2}-{{\Delta }}}\ \hat{a}-\sqrt{\frac{1}{2}+{{\Delta }}}\ \hat{b}\right)+\sqrt{1-{\eta }_{2}}\ {\hat{l}}_{2}\,.$$

After subtraction and amplification, we obtain

27 $$\hat{q}=K{\hat{c}}^{\dagger }\hat{c}-K{\hat{d}}^{\dagger }\hat{d}$$

28 $$=\beta \tilde{g}B+\tilde{g}A{\hat{X}}_{a}+\tilde{g}B{\hat{X}}_{b}+\tilde{g}{L}_{1}{\hat{X}}_{l1}+\tilde{g}{L}_{2}{\hat{X}}_{l2}$$

with $\tilde{g}:= K\beta$. Here we have introduced the quadrature operators

29 $${\hat{X}}_{a}=\hat{a}+{\hat{a}}^{\dagger }\,,$$

30 $${\hat{X}}_{b}=\delta \hat{b}+\delta {\hat{b}}^{\dagger }\,,$$

31 $${\hat{X}}_{l1}={\hat{l}}_{1}+{\hat{l}}_{1}^{\dagger }\,,$$

32 $${\hat{X}}_{l2}={\hat{l}}_{2}+{\hat{l}}_{2}^{\dagger }\,,$$

and the pre-factors are given by

33 $$B=-\left(\frac{1}{2}+{{\Delta }}\right){\eta }_{2}-\left(\frac{1}{2}-{{\Delta }}\right){\eta }_{1}\,,$$

34 $$A=\left({\eta }_{1}+{\eta }_{2}\right)\sqrt{\frac{1}{4}-{{{\Delta }}}^{2}}\,,$$

35 $${L}_{1}=\sqrt{{\eta }_{1}\left(1-{\eta }_{1}\right)\left(\frac{1}{2}-{{\Delta }}\right)}\,,$$

36 $${L}_{2}=\sqrt{{\eta }_{2}\left(1-{\eta }_{2}\right)\left(\frac{1}{2}+{{\Delta }}\right)}\,.$$

The homodyne detection circuit implements a high-pass filter that removes the first term, which is constant. For an ideal homodyne detector, with Δ = 0 and *η*_1_ = *η*_2_ = 1, the output current of the detector reduces to

37 $${\hat{q}}_{0}=\tilde{g}{\hat{X}}_{a}\,.$$

All the other terms that appear in Eq. (28) are treated as noise. We define the noise operator, $\hat{N}=(B{\hat{X}}_{b}+{L}_{1}{\hat{X}}_{l1}+{L}_{2}{\hat{X}}_{l2})/A$, and rewrite Eq. (28) as

38 $$\hat{q}=g\left({\hat{X}}_{a}+\hat{N}\right)$$

with $g=\tilde{g}A$. Note that electronic noise can also be modeled in this way, by attributing it to fluctuations in the auxiliary modes ${\hat{l}}_{1}$ and ${\hat{l}}_{2}$ or in the local oscillator mode $\delta \hat{b}$. The goal of the QRNG system is to extract bits from the measured homodyne output $\hat{q}$, with the requirement that these bits are random with respect to the noisy variable $\hat{N}$. This requirement means that the extracted random bits look random to an agent that has perfect knowledge, not only of the system specifications but also of $\hat{N}$. Note that the noise comes from the fluctuations of the variables ${\hat{X}}_{b}$, ${\hat{X}}_{l1}$, and ${\hat{X}}_{l2}$ and is thus ultimately of quantum nature. For example, an agent may prepare the initial state of the modes ${\hat{l}}_{1}$ and ${\hat{l}}_{2}$ and measure them after the interaction at the beam splitters shown in Fig. 5.

The finite bandwidth of the detector can be modeled by its impulse response *h*_amp_, which is the Fourier transform of its frequency response. The output voltage is then given by

39 $${V}_{\text{out}}(t)=q(t)* {h}_{\text{amp}}(t)\,,$$

where * is a convolution. Electronic noise also has finite bandwidth, and we assume it to have a Gaussian distribution with PSD *S*_elec_(*λ*), zero mean, and variance ${\sigma }_{{\text{elec}}}^{2}=\int_{0}^{2\pi }{S}_{\text{elec}}(\lambda)/2\pi d\lambda$.

In our calibration method, described in the main text, we replace the vacuum state in the signal mode $\hat{a}$ with a coherent state. This allows us to estimate the contribution of the vacuum fluctuations, ${\hat{X}}_{a}$, to the PSD of the detector output.

### Theoretical analysis in the i.i.d. limit

Consider a single optical mode characterized by the quadrature operators $\hat{q}$ and $\hat{p}$. For a thermal state *ρ*_*S*_ with mean photon number *n*, the first moments of the field quadratures vanish, and the covariance matrix (CM) is

40 $${V}_{{\rm{thermal}}}=\left(\begin{array}{ll}\langle {\hat{q}}^{2}\rangle &\frac{1}{2}\langle \hat{q}\hat{p}+\hat{p}\hat{q}\rangle \\ \frac{1}{2}\langle \hat{p}\hat{q}+\hat{q}\hat{p}\rangle &\langle {\hat{p}}^{2}\rangle \end{array}\right)$$

41 $$=\left(\begin{array}{ll}1+2n&0\\ 0&1+2n\end{array}\right)\ ,$$

where we, as a matter of convention, put the variance of the vacuum equal to 1. In the equation above, we use $\langle \hat{O}\rangle := \,{\text{tr}}\,({\rho }_{S}\hat{O})$ for operator $\hat{O}$. For such a state, the output *X* of homodyne detection is distributed according to a Gaussian law,

42 $${p}_{X}(x)=G(x;0,{g}^{2}(1+2n))\ ,$$

where *g* is a gain factor.

As discussed above, the measured state *ρ*_*S*_ is purified into a TMSV. Thereby the second optical mode of this TMSV state, characterized by the field quadratures ${\hat{q}}_{{\rm{e}}}$ and ${\hat{p}}_{{\rm{e}}}$, is associated with the environment, i.e., the rest of the universe. The TMSV state is a Gaussian state with zero mean and CM^24^

43 $$V=\left(\begin{array}{llll}\langle {\hat{q}}^{2}\rangle &\frac{1}{2}\langle \hat{q}\hat{p}+\hat{p}\hat{q}\rangle &\langle \hat{q}{\hat{q}}_{{\rm{e}}}\rangle &\langle \hat{q}{\hat{p}}_{{\rm{e}}}\rangle \\ \frac{1}{2}\langle \hat{p}\hat{q}+\hat{q}\hat{p}\rangle &\langle {\hat{p}}^{2}\rangle &\langle \hat{p}{\hat{q}}_{{\rm{e}}}\rangle &\langle \hat{p}{\hat{p}}_{{\rm{e}}}\rangle \\ \langle {\hat{q}}_{{\rm{e}}}\hat{q}\rangle &\langle {\hat{q}}_{{\rm{e}}}\hat{p}\rangle &\langle {\hat{q}}_{{\rm{e}}}^{2}\rangle &\frac{1}{2}\langle {\hat{q}}_{{\rm{e}}}{\hat{p}}_{{\rm{e}}}+{\hat{p}}_{{\rm{e}}}{\hat{q}}_{{\rm{e}}}\rangle \\ \langle {\hat{p}}_{{\rm{e}}}\hat{q}\rangle &\langle {\hat{p}}_{{\rm{e}}}\hat{p}\rangle &\frac{1}{2}\langle {\hat{p}}_{{\rm{e}}}{\hat{q}}_{{\rm{e}}}+{\hat{q}}_{{\rm{e}}}{\hat{p}}_{{\rm{e}}}\rangle &\langle {\hat{p}}_{{\rm{e}}}^{2}\rangle \end{array}\right)$$

44 $$ =\left(\begin{array}{llll}1+2n&0&2\sqrt{n(n+1)}&0\\ 0&1+2n&0&-2\sqrt{n(n+1)}\\ 2\sqrt{n(n+1)}&0&1+2n&0\\ 0&-2\sqrt{n(n+1)}&0&1+2n\end{array}\right)\ .$$

The correlations between the outcome *X* of ideal homodyne detection and the quantum side information in its environment are described by the CQ state

45 $${\rho }_{XE}=\int {\rm{d}}x\ {p}_{X}(x)\left|x\right\rangle \left\langle x\right|\otimes {\rho }_{E}^{x}\ ,$$

where $\left|x\right\rangle$ are orthogonal states used to represent the possible outcomes of homodyne detection, and the integral in Eq. (45) extends over the real line. The state ${\rho }_{E}^{x}$ is the conditional state of the environment for a given measurement output value *x*. Without loss of generality, we consider the case where the quadrature $\hat{q}$ is measured. We can then compute (see Supplementary Note 1 for details of the derivation) the first moment of the field quadratures of ${\rho }_{E}^{x}$:

46 $$\left(\begin{array}{l}\langle {\hat{q}}_{{\rm{e}}}\rangle \\ \langle {\hat{p}}_{{\rm{e}}}\rangle \end{array}\right)=\left(\begin{array}{l}\frac{2\sqrt{n(n+1)}}{g(1+2n)}x\\ 0\end{array}\right)\ ,$$

as well as the CM

47 $$\left(\begin{array}{ll}\langle {\hat{q}}_{{\rm{e}}}^{2}\rangle &\frac{1}{2}\langle {\hat{q}}_{{\rm{e}}}{\hat{p}}_{{\rm{e}}}+{\hat{p}}_{{\rm{e}}}{\hat{q}}_{{\rm{e}}}\rangle \\ \frac{1}{2}\langle {\hat{p}}_{{\rm{e}}}{\hat{q}}_{{\rm{e}}}+{\hat{q}}_{{\rm{e}}}{\hat{p}}_{{\rm{e}}}\rangle &\langle {\hat{p}}_{{\rm{e}}}^{2}\rangle \end{array}\right)=\left(\begin{array}{ll}\frac{1}{1+2n}&0\\ 0&1+2n\end{array}\right)\ .$$

The continuous variable *X* is mapped into a discrete and bounded variable $\bar{X}$ due to the use of an ADC. The probability mass distribution of $\bar{X}$ is

48 $${p}_{\overline{X}}(j)=\int_{{I}_{j}}{\rm{d}}x \ {p}_{X}(x)\ ,$$

where *I*_*j*_s are *d* intervals that discretize the outcome of homodyne detection. In a typical setting, these *d* non-overlapping intervals *I*_*j*_ are of the form

49 $${I}_{1}=(-\infty ,-R]\ ,$$

50 $${I}_{d}=(R,\infty)\ ,$$

and for *j* = 2, …, *d* − 1

51 $${I}_{j}=({a}_{j}-{{\Delta }}x/2,{a}_{j}+{{\Delta }}x/2]\ ,$$

with *a*_*j*_ = − *R* + (*j* − 1)Δ*x*/2 and Δ*x* = 2*R*/(*d* − 2). This choice of the intervals reflects the way in which an ideal ADC with range *R* and bin size Δ*x* operates in mapping a continuous variable into a discrete one. However, ADCs are not ideal devices, and below we show how the digitization error of the ADC reduces the min-entropy.

In terms of the discrete variable $\bar{X}$, the correlations with the environment are thus described by the state

52 $${\rho }_{\overline{X}E}={\mathop{\sum}\limits_{j}} \ {p}_{\overline{X}}(j)\left|j\right\rangle \left\langle j\right|\otimes {\rho }_{E}^{(j)}\ ,$$

with

53 $${\rho }_{E}^{(j)}=\frac{1}{{p}_{\overline{X}}(j)}\int_{{I}_{j}}{\rm{d}}x \ {p}_{X}(x){\rho }_{E}^{x}\ .$$

We are now ready to quantify the rate of the QRNG in terms of the conditional min-entropy. Given the state ${\rho }_{\overline{X}E}$ in Eq. (52), the min-entropy of $\bar{X}$ conditioned on the eavesdropper (denoted with the letter *E*) reads^28^

54 $${H}_{\min }{(\bar{X}| E)}_{\rho }= {{\mathop{{\mathrm{sup}}}\limits_{{\gamma }_{E}}}} \left[-\mathrm{log}\,\parallel {\gamma }_{E}^{-1/2}{\rho }_{\overline{X}E}\ {\gamma }_{E}^{-1/2}{\parallel }_{\infty }\right]\ ,$$

where ∥⋅∥_*∞*_ denotes the operator norm (equal to the value of the maximum eigenvalue), and the supremum is over a density operator *γ*_*E*_ for the environment system.

Since a direct computation of the min-entropy is not feasible, as it requires an optimization over all density operators *γ*_*E*_ in an infinite-dimensional Hilbert space, we instead focus on finding a computable lower bound. A first lower bound on the min-entropy is obtained by computing $\parallel {\gamma }_{E}^{-1/2}{\rho }_{\overline{X}E}\ {\gamma }_{E}^{-1/2}{\parallel }_{\infty }$ for a given choice of the state *γ*_*E*_, so that we have

55 $${H}_{\min }{(\bar{X}| E)}_{\rho }\ge -\mathrm{log}\,\parallel {\gamma }_{E}^{-1/2}{\rho }_{\overline{X}E}\ {\gamma }_{E}^{-1/2}{\parallel }_{\infty }$$

56 $$=-\mathrm{log}\,\left[{{\mathop{{\mathrm{sup}}}\limits_{j}}}\ {p}_{\overline{X}}(j)\ \parallel {\gamma }_{E}^{-1/2}{\rho }_{E}^{(j)}{\gamma }_{E}^{-1/2}{\parallel }_{\infty }\right]\ ,$$

where the last equality holds because the eigenstates $\left|j\right\rangle$ of ${\rho }_{\overline{X}E}$ in Eq. (52) are mutually orthogonal. Here we set *γ*_*E*_ equal to a Gaussian state with zero mean and CM

57 $$\left(\begin{array}{ll}1+2(n+\delta)&0\\ 0&1+2(n+\delta)\end{array}\right)\ ,$$

where the parameter *δ* will be optimized a posteriori to improve the bound.

A second lower bound is obtained by applying the triangular inequality,

58 $$	{p}_{\overline{X}}(j)\ \parallel {\gamma }_{E}^{-1/2}{\rho }_{E}^{(j)}{\gamma }_{E}^{-1/2}{\parallel }_{\infty }\\ 	=\,\parallel {\gamma }_{E}^{-1/2}\int_{{I}_{j}}dx\ {p}_{X}(x)\ {\rho }_{E}^{x}{\gamma }_{E}^{-1/2}{\parallel }_{\infty }$$

59 $$\;\;\;\;\le\! \int_{{I}_{j}}{\rm{d}}x\ {p}_{X}(x)\ \parallel {\gamma }_{E}^{-1/2}{\rho }_{E}^{x}\ {\gamma }_{E}^{-1/2}{\parallel }_{\infty }\ ,$$

which implies

60 $${H}_{\min }(\bar{X}| E)\ge -\mathrm{log}\,\left[{{\mathop{{\mathrm{sup}}}\limits_{j}}}\int_{{I}_{j}}{\rm{d}}x\ {p}_{X}(x)\ \parallel {\gamma }_{E}^{-1/2}{\rho }_{E}^{x}\ {\gamma }_{E}^{-1/2}{\parallel }_{\infty }\right]\ .$$

Since ${\rho }_{E}^{x}$ and *γ*_*E*_ are both Gaussian states, the above lower bound can be computed using the Gibbs representation techniques developed in ref. ^36^. Employing these techniques and additional tools, ref. ^37^ derived a formula for the min-entropy. By applying this result, we obtain (see Supplementary Note 2 for details)

61 $$	\int_{{I}_{j}}{\rm{d}}x\ {p}_{X}(x)\ \parallel {\gamma }_{E}^{-1/2}{\rho }_{E}^{x}{\gamma }_{E}^{-1/2}{\parallel }_{\infty }\\ 	\quad=\frac{1}{g}\frac{(n+\delta)(1+n+\delta)}{\sqrt{2\pi \delta (2n(n+1+\delta)+\delta)}}\int_{{I}_{j}}{\rm{d}}x\ \exp \left[\frac{-{x}^{2}}{2{g}^{2}}\frac{\delta }{2n(n+1+\delta)+\delta }\right]\ .$$

To simplify the notation, we define

62 $$g^{\prime} := g\sqrt{\frac{4n(n+1+\delta)+2\delta }{\delta }}\ .$$

This yields

63 $$\int_{{I}_{j}}{\rm{d}}x\ {p}_{X}(x)\ \parallel {\gamma }_{E}^{-1/2}{\rho }_{E}^{x}{\gamma }_{E}^{-1/2}{\parallel }_{\infty }=\frac{(n+\delta)(1+n+\delta)}{\delta g^{\prime} \sqrt{\pi }}\int_{{I}_{j}}{\rm{d}}x\ \exp \left(\frac{-{x}^{2}}{{g^{\prime} }^{2}}\right)\ .$$

For *j* = 2, …, *d* − 1, this latter quantity reads

64 $$	\int_{{I}_{j}}{\rm{d}}x\ {p}_{X}(x)\ \parallel {\gamma }_{E}^{-1/2}{\rho }_{E}^{x}{\gamma }_{E}^{-1/2}{\parallel }_{\infty } \\ 	\quad=\frac{(n+\delta)(1+n+\delta)}{2\delta }\left[{\rm{erf}}\left(\frac{{a}_{j}}{g^{\prime} }+\frac{{{\Delta }}x}{2g^{\prime} }\right)-{\rm{erf}}\left(\frac{{a}_{j}}{g^{\prime} }-\frac{{{\Delta }}x}{2g^{\prime} }\right)\right]$$

65 $$\le \frac{(n+\delta)(1+n+\delta)}{\delta }\ {\rm{erf}}\left(\frac{{{\Delta }}x}{2g^{\prime} }\right)\ ,$$

and for *j* = 1 and *j* = *d*,

66 $$\int_{{I}_{j}}{\rm{d}}x\ {p}_{X}(x)\ \parallel {\gamma }_{E}^{-1/2}{\rho }_{E}^{x}{\gamma }_{E}^{-1/2}{\parallel }_{\infty }=\frac{(n+\delta)(1+n+\delta)}{2\delta }\ {\rm{erfc}}\left(\frac{R}{g^{\prime} }\right)\ .$$

We hence obtain

67 $${H}_{\min }(\bar{X}| E)\ge -{\mathrm{log}}\,\left[\frac{(n+\delta)(1+n+\delta)}{\delta }\max \left\{{\rm{erf}}\left(\frac{{{\Delta }}x}{2g^{\prime} }\right),\frac{1}{2}{\rm{erfc}}\left(\frac{R}{g^{\prime} }\right)\right\}\right]\ .$$

We remark that this is in fact a family of lower bounds parameterized by *δ* and *g*. The best bound in the family is

68 $${H}_{\min }(\bar{X}| E)\ge	 -\mathop{\min }\limits_{\delta }\mathrm{log}\,\left[\frac{(n+\delta)(1+n+\delta)}{\delta }\right]\\ 	-\mathop{\min }\limits_{g^{\prime} }{\mathrm{log}}\,\left[\max \left\{{\rm{erf}}\left(\frac{{{\Delta }}x}{2g^{\prime} }\right),\frac{1}{2}{\rm{erfc}}\left(\frac{R}{g^{\prime} }\right)\right\}\right]$$

69 $$=-{\mathrm{log}}\,{\left(\sqrt{n}+\sqrt{n+1}\right)}^{2}-{\mathrm{log}}\,\left[\mathop{\min }\limits_{g^{\prime} }\max \left\{{\rm{erf}}\left(\frac{{{\Delta }}x}{2g^{\prime} }\right),\frac{1}{2}{\rm{erfc}}\left(\frac{R}{g^{\prime} }\right)\right\}\right]\ .$$

Let us define the function

70 $$Q(g):= \mathop{\min }\limits_{g^{\prime} }\max \left\{{\rm{erf}}\left(\frac{{{\Delta }}x}{2g^{\prime} }\right),\frac{1}{2}{\rm{erfc}}\left(\frac{R}{g^{\prime} }\right)\right\}\ .$$

Note that ${\rm{erf}}\left(\frac{{{\Delta }}x}{2g^{\prime} }\right)$ is a monotonically decreasing function of $g^{\prime}$ with values in [0, 1), whereas $\frac{1}{2}{\rm{erfc}}\left(\frac{R}{g^{\prime} }\right)$ is monotonically increasing with values in [0, 1/2). This implies that there exists a unique value of ${g}_{* }^{\prime}$ such that

71 $${\rm{erf}}\left(\frac{{{\Delta }}x}{2{g}_{* }^{\prime}}\right)=\frac{1}{2}{\rm{erfc}}\left(\frac{R}{{g}_{* }^{\prime}}\right)\ .$$

If $g^{\prime} \, > \, {g}_{* }^{\prime}$, then $Q(g^{\prime})={\rm{erf}}\left(\frac{{{\Delta }}x}{2g^{\prime} }\right) \, > \, Q({g}_{* }^{\prime})$, and if $g^{\prime} \, < \, {g}_{* }^{\prime}$, then $Q(g^{\prime})=\frac{1}{2}{\rm{erfc}}\left(\frac{R}{g^{\prime} }\right) \, > \, Q({g}_{* }^{\prime})$. This implies that ${g}_{* }^{\prime}$ is a local and global maximum for the function *Q*.

In conclusion, the best lower bound on the conditional min-entropy is

72 $${H}_{\min }(\bar{X}| E)\ge -\mathrm{log}\,{\left(\sqrt{n}+\sqrt{n+1}\right)}^{2}-\mathrm{log}\,\left[{\rm{erf}}\left(\frac{{{\Delta }}x}{2{g}_{* }^{\prime}}\right)\right]\ ,$$

with ${g}_{* }^{\prime}$ implicitly given in Eq. (71).

### ADC digitization noise

ADCs are not ideal devices and are subject to digitization error. We model the digitization error by introducing:

1. A classical noise variable *N*, with associated probability distribution *p*_*N*_;
2. A function *f* that describes how the noise variable *i* combines with the noiseless output value *j* to produce the noisy output *f* = *f*(*j*, *i*).

Using this model, the quantum side information about the output of the noisy ADC is described by the CQ state

73 $${\rho }_{\overline{X}EN}={\mathop{\sum} \limits_{ji}} \ {p}_{\overline{X}}(j)\left|f(j,i)\right\rangle \left\langle f(j,i)\right|\otimes {\rho }_{j}\otimes {p}_{N}(i)\left|i\right\rangle \left\langle i\right|\ ,$$

where we have introduced a dummy quantum register *N* to keep track of the noise value *i*.

We want to ensure that the randomness extracted is also independent on the noise variable *N*, therefore, we compute the min-entropy conditioned on *EN*,

74 $${H}_{\min }(\hat{X}| EN)\ge -{\mathrm{log}}\,\left[{\left\Vert {\gamma }_{EN}^{-1/2}{\rho }_{\overline{X}EN}{\gamma }_{EN}^{-1/2}\right\Vert }_{\infty }\right]$$

75 $$=-{\mathrm{log}}\,\left[{\left\Vert {\mathop{\sum}\limits_{ji}} \ {p}_{\overline{X}}(j){p}_{N}(j)\left|f(j,i)\right\rangle \left\langle f(j,i)\right|\otimes {\gamma }_{EN}^{-1/2}{\rho }_{j}\otimes \left|i\right\rangle \left\langle i\right|{\gamma }_{EN}^{-1/2}\right\Vert }_{\infty }\right]$$

76 $$=-\mathrm{log}\,\left[{{\mathop{{\mathrm{sup}}}\limits_{f}}}{\left\Vert {\mathop{\sum}\limits_{ji\in {S}_{f}}}{p}_{\overline{X}}(j){p}_{N}(i){\gamma }_{EN}^{-1/2}{\rho }_{j}\otimes \left|i\right\rangle \left\langle i\right|{\gamma }_{EN}^{-1/2}\right\Vert }_{\infty }\right]\ ,$$

where *S*_*f*_ denotes the set of values of *j*, *i* such that *f*(*j*, *i*) = *f*.

Putting ${\gamma }_{EN}={\gamma }_{E}\otimes {\sum }_{i}{p}_{N}(i)\left|i\right\rangle \left\langle i\right|$, we obtain

77 $${H}_{\min }(\hat{X}| EN)\ge -\mathrm{log}\,\left[{{\mathop{{\mathrm{sup}}}\limits_{f}}}{\left\Vert {\mathop{\sum} \limits_{ji\in {S}_{f}}}{p}_{\overline{X}}(j){\gamma }_{E}^{-1/2}{\rho }_{j}{\gamma }_{E}^{-1/2}\otimes \left|i\right\rangle \left\langle i\right|\right\Vert }_{\infty }\right]$$

78 $$\ge -\mathrm{log}\,\left[{{\mathop{{\mathrm{sup}}}\limits_{f,i}}}{\left\Vert {\mathop{\sum}\limits_{j\in {S}_{f| i}}}{p}_{\overline{X}}(j){\gamma }_{E}^{-1/2}{\rho }_{j}{\gamma }_{E}^{-1/2}\right\Vert }_{\infty }\right]\ ,$$

where *S*_*f*∣*i*_ is defined as the set of values of *j* such that *f*(*j*, *i*) = *f* for a given value of *i*. We further define *J*_*f*_ as the set of values of *j* such that *f*(*j*, *i*) = *f* for some value of *i*.

It is difficult to estimate *S*_*f*∣*i*_ without making further assumptions on the noise underlying the ADC. However, we can experimentally estimate the cardinality ∣*J*_*f*_∣ of the set *J*_*f*_. Note that *J*_*f*_ contains *S*_*f*∣*i*_ for all *i*. We can then write a computable bound in terms of ∣*J*_*f*_∣:

79 $${H}_{\min }(\hat{X}| EN)\ge -{\mathrm{log}}\,\left[{{\mathop{{\mathrm{sup}}}\limits_{f}}}{\left\Vert {\mathop{\sum}\limits_{j\in {J}_{f}}}\ {p}_{\overline{X}}(j){\gamma }_{E}^{-1/2}{\rho }_{j}{\gamma }_{E}^{-1/2}\right\Vert }_{\infty }\right]$$

80 $$\ge -{\mathrm{log}}\,\left[{{\mathop{{\mathrm{sup}}}\limits_{f}}}{\mathop{\sum}\limits_{j\in {J}_{f}}} \ {p}_{\overline{X}}(j)\ {\left\Vert {\gamma }_{E}^{-1/2}{\rho }_{j}{\gamma }_{E}^{-1/2}\right\Vert }_{\infty }\right]$$

81 $$\ge -{\mathrm{log}}\,\left[{{\mathop{{\mathrm{sup}}}\limits_{f}}}| {J}_{f}| \ {{\mathop{{\mathrm{sup}}}\limits_{j\in {J}_{f}}}} \ {p}_{\overline{X}}(j)\ {\left\Vert {\gamma }_{E}^{-1/2}{\rho }_{j}{\gamma }_{E}^{-1/2}\right\Vert }_{\infty }\right]$$

82 $$\ge -{\mathrm{log}}\,\left[{{\mathop{{\mathrm{sup}}}\limits_{f}}}| {J}_{f}| \ {{\mathop{{\mathrm{sup}}}\limits_{j}}} \ {p}_{\overline{X}}(j) {\left\Vert {\gamma }_{E}^{-1/2}{\rho }_{j}{\gamma }_{E}^{-1/2}\right\Vert }_{\infty }\right]$$

83 $$=-{\mathrm{log}}\,\left[{{\mathop{{\mathrm{sup}}}\limits_{j}}} \ {p}_{\overline{X}}(j)\ {\left\Vert {\gamma }_{E}^{-1/2}{\rho }_{j}{\gamma }_{E}^{-1/2}\right\Vert }_{\infty }\right]-{\mathrm{log}}\,\left[{{\mathop{{\mathrm{sup}}}\limits_{f}}}| {J}_{f}| \right]\ .$$

Here the first inequality follows from the fact that *J*_*f*_ contains *S*_*f*∣*i*_ for all *i*; the second inequality follows from the triangular inequality; the third inequality follows from the fact that the supremum is larger than the average; and the fourth inequality is obtained by replacing the supremum over *j* ∈ *J*_*f*_ with the supremum over all values of *j*.

In conclusion, when compared with an ideal noiseless ADC, the randomness is reduced by at most *b* bits, with $b=\mathrm{log}\,\left[{\sup }_{f}| {J}_{f}| \right]$.

### Verification of assumptions in the theoretical analysis

An integral part is the verification that our implementation indeed fulfills the assumptions made in the theoretical analysis of the QRNG.

#### A1

The physical model above verifies that our detector indeed performs homodyne detection.

The condition of the measurement of a single mode are given due to the following arguments: The local oscillator laser has a side-mode suppression of >70 dB and therefore operates in a single frequency mode. The local oscillator furthermore defines the polarization and the spatial properties (given by the single mode fiber) of the measured mode. The temporal properties are given by the impulse response of the homodyne detector and the following electronic circuits.

The linearity of our detector has been tested by connecting the output to an electrical spectrum analyzer instead of the ADC. Varying the power of the signal laser in the TF calibration set-up, see Fig. 3, we verified its linear operation. We note that the linearity of the output of the homodyne detection circuit before it is sampled by the ADC is the important figure of merit. Nonlinearities introduced by the ADC are taken into account separately by the ADC characterization.

#### A2

The excess noise in the thermal state stems from relative intensity noise of the laser and the electronic noise of the homodyne circuit. Both are independent of the phase between local oscillator and the measured quantum state and can therefore be modeled as phase invariant state.

**Fig. 6:**
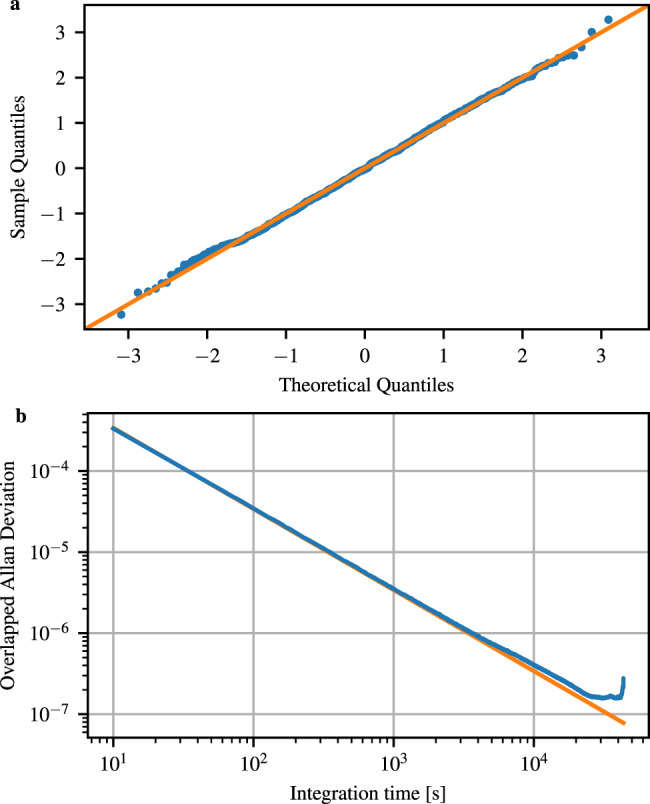
Verification of assumption A2. **a** Quantile–quantile plot indicating the Gaussianity of the measured samples. The variance of the samples has been normalized to 1. The limited analog-to-digital converter range truncates the tails of the Gaussian distribution, which results in slight deviations from the theoretical quantiles toward the ends. **b** Overlapped Allan deviation of vacuum state measurements. The stationarity condition is fulfilled when the experimental points follow the theory curve, which is the case until about 1000 s where it starts to deviate.

Having established the phase invariance of the measured state, we verify the Gaussianity of the measured signal. This can only be shown approximately and is displayed in Fig. 6a where we show the probability quantiles of the measured samples and compared those to the theoretical quantiles of a Gaussian distribution. This completes the verification of the assumption in the security proof that a thermal state is measured.

We are left with that the mean photon number of the thermal state shall be stationary. Also this can only be proven approximately. We computed the overlapped Allan deviation of the measurement outcomes, which is shown in Fig. 6b. It is clearly visible that in the short term the noise processes are stationary. Over longer times, some fluctuations become evident, which could lead to a lower min-entropy at times than estimated. A power stabilization of the local oscillator laser could improve this figure of merit. We, however, leave this investigation for future work.

### Real-time randomness extraction

Having calculated the min-entropy, the next step is to extract random numbers. This is done by using a strong extractor based on a Toeplitz matrix hashing algorithm in which the seed can be reused^38^. We chose matrix dimensions of *n* = 5632 bits and *m* = 1024 bits, which corresponds to 352 input samples with a depth of 16 bit and an output length *m* < *l*, chosen such that Eq. (1) was fulfilled with ${H}_{\min }=3.51$ bit and *ϵ*_hash_ < 10^−32^. The 16-bit samples provided by the ADC at a rate of 1 GHz are received by the FPGA in chunks of 64 bits at a rate of 250 MHz. For the algorithm implementing the Toeplitz hashing, we followed the approach of ref. ^20^. Every clock cycle 64 bits were stored in a block until *n*-bits were accepted, after which the next block started receiving data. For each full block, we carried out the hashing multiplication with bit-wise AND and subsequent XOR operations on the Toeplitz matrix by first splitting up the matrix into submatrices of width 16 bit and then shifting the data through the operations. When the hashing was completed, the *m*-bit-wide output data was stored in a register, and the next block was processed. The achieved throughput was 2.9 Gbit/s.

### Reporting summary

Further information on research design is available in the Nature Research Reporting Summary linked to this article.

## Supplementary information

Supplementary Information

Reporting Summary

## Data Availability

All experimental data are available from the authors upon reasonable request.

## Code availability

All codes are available from the authors upon reasonable request.
