# Supplementary material for: Homodyne-based quantum random number generator at 2.9 Gbps secure against quantum side-information
Source: Nat Commun. 2021 Jan 27;12:605. doi: 10.1038/s41467-020-20813-w (PMC7840728; doi:10.1038/s41467-020-20813-w)
Supplement: Supplementary file 1 — Supplementary Information [file 41467_2020_20813_MOESM1_ESM.pdf]

Supplemental Material: Homodyne-based quantum random number generator at 2.9 Gbps secure against quantum side-information

Tobias Gehring,[1,*] Cosmo Lupo,[2,3] Arne Kordts,[1] Dino Solar Nikolic,[1] Nitin Jain,[1]
Tobias Rydberg,[1] Thomas B. Pedersen,[4] Stefano Pirandola,[2] and Ulrik L. Andersen[1,†]

[1]*Center for Macroscopic Quantum States (bigQ), Department of Physics, Technical University of Denmark, Fysikvej, 2800 Kgs. Lyngby, Denmark*
[2]*Department of Computer Science, University of York, York YO10 5GH, United Kingdom*
[3]*Department of Physics and Astronomy, University of Sheffield, United Kingdom*
[4]*Cryptomathic A/S, Jaegergardsgade 118, 8000 Aarhus C, Denmark*

In this appendix we present a number of technical results: (1) we calculate the moments of the conditional quantum state, (2) we calculate the conditional min-entropy for Gaussian states, (3) we describe the ADC characterization, (4) we discuss the estimation of the noise variance and entropy rate from experimental data, (5) we describe the characterization of the power spectral density of the vacuum fluctuations, and (6) we describe as an application example the requirements on QRNGs when used in continuous-variable quantum key distribution.

* tobias.gehring@fysik.dtu.dk
† ulrik.andersen@fysik.dtu.dk

I. SUPPLEMENTARY NOTE 1: CALCULATION OF THE MOMENTS OF THE CONDITIONAL QUANTUM STATE

Consider a system of two bosonic modes, where the first mode has canonical (phase and quadrature) operators $\hat{q}$, $\hat{p}$, and the second mode has canonical operators $\hat{q}_e$, $\hat{p}_e$. If the two modes are in a Two-Mode Squeezed Vacuum (TMSV) state, the Covariance Matrix (CM) reads [1]

$$V = \left(\begin{array}{c|c} A & C \\ \hline C^{\mathsf{T}} & B \end{array}\right) = \left(\begin{array}{cc|cc} \langle \hat{q}^2 \rangle & \frac{1}{2}\langle \hat{q}\hat{p} + \hat{p}\hat{q} \rangle & \langle \hat{q}\hat{q}_e \rangle & \langle \hat{q}\hat{p}_e \rangle \\ \frac{1}{2}\langle \hat{p}\hat{q} + \hat{q}\hat{p} \rangle & \langle \hat{p}^2 \rangle & \langle \hat{p}\hat{q}_e \rangle & \langle \hat{p}\hat{p}_e \rangle \\ \hline \langle \hat{q}_e\hat{q} \rangle & \langle \hat{q}_e\hat{p} \rangle & \langle \hat{q}_e^2 \rangle & \frac{1}{2}\langle \hat{q}_e\hat{p}_e + \hat{p}_e\hat{q}_e \rangle \\ \langle \hat{p}_e\hat{q} \rangle & \langle \hat{p}_e\hat{p} \rangle & \frac{1}{2}\langle \hat{p}_e\hat{q}_e + \hat{q}_e\hat{p}_e \rangle & \langle \hat{p}_e^2 \rangle \end{array}\right) \tag{1}$$

$$= \left(\begin{array}{cc|cc} 1+2n & 0 & 2\sqrt{n(n+1)} & 0 \\ 0 & 1+2n & 0 & -2\sqrt{n(n+1)} \\ \hline 2\sqrt{n(n+1)} & 0 & 1+2n & 0 \\ 0 & -2\sqrt{n(n+1)} & 0 & 1+2n \end{array}\right). \tag{2}$$

The inverse matrix of the CM of the TMSV is

$$M = V^{-1} = \left(\begin{array}{c|c} A & -C \\ \hline -C^{\mathsf{T}} & B \end{array}\right) = \left(\begin{array}{cc|cc} 1+2n & 0 & -2\sqrt{n(n+1)} & 0 \\ 0 & 1+2n & 0 & 2\sqrt{n(n+1)} \\ \hline -2\sqrt{n(n+1)} & 0 & 1+2n & 0 \\ 0 & 2\sqrt{n(n+1)} & 0 & 1+2n \end{array}\right). \tag{3}$$

We can express this state using the Wigner function. Putting $\boldsymbol{r} = (q, p, q_e, p_e)^{\mathsf{T}}$, the Wigner function of the TMSV is

$$W(q,p,q_e,p_e) = \frac{1}{(2\pi)^2}\exp\left[-\frac{1}{2}\boldsymbol{r}^{\mathsf{T}}V^{-1}\boldsymbol{r}\right] = \frac{1}{(2\pi)^2}\exp\left[-\frac{1}{2}\boldsymbol{r}^{\mathsf{T}}M\boldsymbol{r}\right] \tag{4}$$

$$= \frac{1}{(2\pi)^2}\exp\left[-\left(\frac{1}{2}+n\right)\left(q^2+p^2+q_e^2+p_e^2\right) - 2\sqrt{n(n+1)}\,(pp_e - qq_e)\right]. \tag{5}$$

The first mode is measured by applying ideal homodyne detection. The probability density that a measurement of the quadrature $\hat{q}$ outputs the value q_0 can be computed directly from the Wigner function:

$$P(q_0) = \int dq\,dp\,dq_e\,dp_e W(q,p,q_e,p_e)\delta(q-q_0) = \frac{1}{\sqrt{2\pi}\sqrt{1+2n}}\exp\left[-\frac{q_0^2}{2(1+2n)}\right] \tag{6}$$

Given the measurement output q_0, the conditional Wigner function of the second mode reads

$$W(q_e,p_e|q_0) = \frac{1}{P(q_0)}\int dq\,dp W(q,p,q_e,p_e)\delta(q-q_0) \tag{7}$$

$$= \frac{1}{P(q_0)(2\pi)^2}\int dp\exp\left[-\left(\frac{1}{2}+n\right)\left(q_0^2+p^2+q_e^2+p_e^2\right) - 2\sqrt{n(n+1)}\,(pp_e - q_0q_e)\right] \tag{8}$$

$$= \frac{\exp\left[-\left(\frac{1}{2}+n\right)q_0^2\right]}{P(q_0)(2\pi)^2}\exp\left[-\left(\frac{1}{2}+n\right)q_e^2 + 2\sqrt{n(n+1)}q_0q_e\right]\exp\left[-\left(\frac{1}{2}+n\right)p_e^2\right]$$
$$\times\int dp\exp\left[-\left(\frac{1}{2}+n\right)p^2 - 2\sqrt{n(n+1)}pp_e\right]. \tag{9}$$

The Gaussian integral can be computed by completing the square:

$$\int dp\exp\left[-\left(\frac{1}{2}+n\right)p^2 - 2\sqrt{n(n+1)}pp_e\right] = \exp\left[\frac{2n(n+1)}{1+2n}p_e^2\right]\sqrt{\frac{2\pi}{1+2n}}. \tag{10}$$

Also note that

$$\exp\left[-\left(\frac{1}{2}+n\right)q_e^2 + 2\sqrt{n(n+1)}q_0q_e\right] = \exp\left[\frac{2n(n+1)}{1+2n}q_0^2\right]\exp\left[-\left(q_e\sqrt{\frac{1}{2}+n} - q_0\sqrt{\frac{2n(n+1)}{1+2n}}\right)^2\right] \tag{11}$$

$$= \exp\left[\frac{2n(n+1)}{1+2n}q_0^2\right]\exp\left[-\frac{1}{2}(1+2n)\left(q_e - q_0\frac{2\sqrt{n(n+1)}}{1+2n}\right)^2\right]. \tag{12}$$

Coming back to the Wigner function of the conditional state, we obtain

$$W(q_e,p_e|q_0) = \sqrt{\frac{2\pi}{1+2n}}\frac{\exp\left[-\left(\frac{1}{2}+n\right)q_0^2+\frac{2n(n+1)}{1+2n}q_0^2\right]}{P(q_0)(2\pi)^2}\exp\left[-\frac{1}{2}\left(1+2n\right)\left(q_e-q_0\frac{2\sqrt{n(n+1)}}{1+2n}\right)^2\right]$$
$$\times\exp\left[-\left(\frac{1}{2}+n\right)p_e^2+\frac{2n(n+1)}{1+2n}p_e^2\right] \quad (13)$$
$$= \sqrt{\frac{2\pi}{1+2n}}\frac{\exp\left[-\frac{1}{2}\frac{q_0^2}{1+2n}\right]}{P(q_0)(2\pi)^2}\exp\left[-\frac{1}{2}\left(1+2n\right)\left(q_e-q_0\frac{2\sqrt{n(n+1)}}{1+2n}\right)^2\right]\exp\left[-\frac{1}{2}\frac{p_e^2}{1+2n}\right] \quad (14)$$
$$= \frac{1}{2\pi}\exp\left[-\frac{1}{2}\left(1+2n\right)\left(q_e-q_0\frac{2\sqrt{n(n+1)}}{1+2n}\right)^2\right]\exp\left[-\frac{1}{2}\frac{p_e^2}{1+2n}\right]. \quad (15)$$

We recognise that this is the Wigner function of a Gaussian state with first moments

$$\begin{pmatrix}\langle\hat{q}_e\rangle\\ \langle\hat{p}_e\rangle\end{pmatrix} = \begin{pmatrix}q_0\frac{2\sqrt{n(n+1)}}{1+2n}\\ 0\end{pmatrix}, \quad (16)$$

and CM

$$V_c = \begin{pmatrix}1+2n & 0\\ 0 & \frac{1}{1+2n}\end{pmatrix}^{-1} = \begin{pmatrix}\frac{1}{1+2n} & 0\\ 0 & 1+2n\end{pmatrix}. \quad (17)$$

Finally, if a gain factor g is applied to the output of the homodyne detection, yielding $x = gq_0$, then the first moment becomes

$$\begin{pmatrix}\langle\hat{q}_e\rangle\\ \langle\hat{p}_e\rangle\end{pmatrix} = \begin{pmatrix}x\frac{2\sqrt{n(n+1)}}{g(1+2n)}\\ 0\end{pmatrix}. \quad (18)$$

II. SUPPLEMENTARY NOTE 2: CONDITIONAL MIN-ENTROPY FOR GAUSSIAN STATES

In this Section we present the detailed calculations to obtain our expression of the conditional min-entropy starting from Theorem 24 of Ref. [2]. We start by recalling some well-known facts about Gaussian states of a single bosonic mode. A bosonic mode is identified by the quadrature operators $\hat{q}$, $\hat{p}$. A Gaussian state ρ is uniquely determined by the vector of first moments

$$\boldsymbol{\mu_\rho} = \begin{pmatrix}\langle\hat{q}\rangle\\ \langle\hat{p}\rangle\end{pmatrix} \quad (19)$$

and the CM of the quadrature operators:

$$V_\rho = \begin{pmatrix}\langle(\hat{q}-\langle\hat{q}\rangle)^2\rangle & \frac{1}{2}\langle(\hat{q}-\langle\hat{q}\rangle)(\hat{p}-\langle\hat{p}\rangle)+(\hat{p}-\langle\hat{p}\rangle)(\hat{q}-\langle\hat{q}\rangle)\rangle\\ \frac{1}{2}\langle(\hat{q}-\langle\hat{q}\rangle)(\hat{p}-\langle\hat{p}\rangle)+(\hat{p}-\langle\hat{p}\rangle)(\hat{q}-\langle\hat{q}\rangle)\rangle & \langle(\hat{p}-\langle\hat{p}\rangle)^2\rangle\end{pmatrix}. \quad (20)$$

The symplectic two-form is represented by the matrix

$$\Omega = \begin{pmatrix}0 & 1\\ -1 & 0\end{pmatrix}. \quad (21)$$

The partition function of the state ρ is

$$Z_\rho = \sqrt{\det\left[\frac{1}{2}\left(V_\rho + i\Omega\right)\right]}. \quad (22)$$

Theorem 24 of Ref. [2] established that, for any pair of Gaussian states ρ, γ such that $V_\gamma - V_\rho > 0$, the following holds:

$$\ln \|\rho^{1/2}\gamma^{-1}\rho^{1/2}\|_\infty = \ln\left(\frac{Z_\gamma}{Z_\rho}\right) - \frac{1}{2}\mathrm{Tr}\left\{\mathrm{arcoth}\left(\sqrt{-V'\Omega V'\Omega}\right)\right\} + \frac{1}{2}\boldsymbol{\Delta\mu}^\mathsf{T}\left(V_\gamma - V_\rho\right)^{-1}\boldsymbol{\Delta\mu}\,, \tag{23}$$

where $\ln$ denotes the natural logarithm,

$$V' = V_\rho + \sqrt{I + (V_\rho\Omega)^{-2}}\,V_\rho\left(V_\gamma - V_\rho\right)^{-1}V_\rho\sqrt{I + (\Omega V_\rho)^{-2}}\,, \tag{24}$$

with I denoting the identity matrix, and

$$\boldsymbol{\Delta\mu} = \boldsymbol{\mu_\rho} - \boldsymbol{\mu_\gamma}\,. \tag{25}$$

Equation (23) differs from the statement of Theorem 24 of Ref. [2] by a factor $1/2$ in the last term. This is due to a difference between our definition of the CM [in Eq. (20)] and that given in Eq. (15) of Ref. [2].

Note that we are interested in computing the quantity $\|\gamma^{-1/2}\rho\gamma^{-1/2}\|_\infty$, while the above theorem deals with $\|\rho^{1/2}\gamma^{-1}\rho^{1/2}\|_\infty$. However, it is easy to show that these two norms are equal. In fact, consider the operator $\rho^{1/2}\gamma^{-1/2}$, and its singular value decomposition $\rho^{1/2}\gamma^{-1/2} = UDV$, where U and V are unitary and D is diagonal. We then have $\rho^{1/2}\gamma^{-1}\rho^{1/2} = UD^2U^\dagger$. Analogously, we also have $\gamma^{-1/2}\rho\gamma^{-1/2} = V^\dagger D^2 V$. As the operators $\rho^{1/2}\gamma^{-1}\rho^{1/2}$ and $\gamma^{-1/2}\rho\gamma^{-1/2}$ are unitary equivalent, they do have the same norm.

We put:

$$V_\rho = \begin{pmatrix} \frac{1}{1+2n} + \epsilon & 0 \\ 0 & 1+2n \end{pmatrix}\,, \tag{26}$$

$$\boldsymbol{\mu_\rho} = \begin{pmatrix} x\frac{2\sqrt{n(n+1)}}{g(1+2n)} \\ 0 \end{pmatrix}\,, \tag{27}$$

for $\epsilon > 0$, and

$$V_\gamma = \begin{pmatrix} 1+2(n+\delta) & 0 \\ 0 & 1+2(n+\delta) \end{pmatrix}\,, \tag{28}$$

$$\boldsymbol{\mu_\gamma} = \begin{pmatrix} 0 \\ 0 \end{pmatrix}\,. \tag{29}$$

We thus obtain, in the limit of $\epsilon \to 0$,

$$\ln \|\rho^{1/2}\gamma^{-1}\rho^{1/2}\|_\infty = \ln \|\gamma^{-1/2}\rho\gamma^{-1/2}\|_\infty \tag{30}$$

$$= \frac{1}{2}\ln\left[\frac{(1+2n)(n+\delta)^2(1+n+\delta)^2}{\delta(2n(1+n+\delta)+\delta)}\right] + \frac{n(n+1)}{2n(1+n+\delta)+\delta}\frac{x^2}{g^2(1+2n)}\,. \tag{31}$$

That is,

$$\|\gamma^{-1/2}\rho\gamma^{-1/2}\|_\infty = (n+\delta)(1+n+\delta)\sqrt{\frac{1+2n}{\delta(2n(1+n+\delta)+\delta)}}\,\exp\left[\frac{n(n+1)}{2n(1+n+\delta)+\delta}\frac{x^2}{g^2(1+2n)}\right]. \tag{32}$$

Recall that

$$p_X(x) = G(x;0,g^2(1+2n)) = \frac{1}{g\sqrt{2\pi(1+2n)}}\,e^{-\frac{x^2}{2g^2(1+2n)}}\,, \tag{33}$$

which yields

$$p_X(x)\|\gamma^{-1/2}\rho\gamma^{-1/2}\|_\infty = \frac{1}{g}\frac{(n+\delta)(1+n+\delta)}{\sqrt{2\pi\delta(2n(1+n+\delta)+\delta)}}\,\exp\left[-\frac{x^2}{2g^2}\frac{\delta}{2n(1+n+\delta)+\delta}\right]. \tag{34}$$

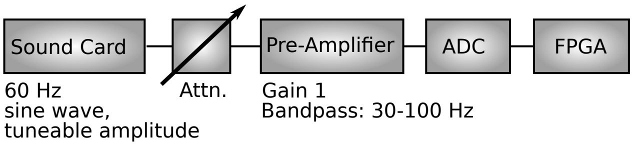

FIG. 1. **Measurement setup for ADC characterization.** The sound card has a high impedance output which was converted to 50 Ohm by the pre-amplifier.

III. SUPPLEMENTARY NOTE 3: ADC CHARACTERIZATION

The setup for the ADC characterization is shown in Fig. 1. A sine wave of 60 Hz frequency was generated by a 24 bit sound card. The maximum amplitude of the sine was adjusted by a variable attenuator to match the input range of the ADC. A pre-amplifier (Standard Research Systems SR560) was used to bandpass filter the signal and as buffer to convert the high impedance output of the sound card to a 50 Ohm impedance as required by the ADC.

The measurement was performed as follows: We swept the amplitude of the sine wave from 0 to $2^{23}-1$ in steps of 64. This yields 4 measurements with different voltage levels for each bin of the 16 bit ADC. For each amplitude setting, 10 periods of the sine wave were recorded by the ADC with a sampling rate of 1 GS/s, and the data was transferred to a computer via the FPGA. We analysed the data by determining the location of the maxima and minima and for each we calculated a histogram from 50,000 samples around the maximum and minimum, respectively. The 10 histograms were summed so that we obtained a histogram from 500,000 samples. To obtain the final histograms we combined the 4 measurements per ADC bin and normalized the probability distribution.

IV. SUPPLEMENTARY NOTE 4: ESTIMATION OF VARIANCES AND THE ENTROPY RATE

In this appendix we discuss the estimation of the variance, entropy rate and conditional variance of the noise and signal. To make things more concrete, we focus on the estimation of the signal variance σ^2, the entropy rate $h(X)$, and the conditional signal variance σ^2_X. Assume that T is the runtime of the experiment, and n signal measurements are performed at regular time intervals of $\delta t = T/n$. The spectral density computed from these data is a function of n discrete frequencies, denoted as ω_j's, taking values between $2\pi/T$ and $2\pi n/T$. Below we work with the discrete variable λ_j defined as $\lambda_j \equiv T\omega_j/n$, which can be approximated by the continuous variable λ taking values with domain $[0, 2\pi]$.

We estimate the spectral density $f(\lambda)$ by applying the Welch's method, according to which the data are first divided in M (possibly overlapping) blocks, and then in each block the periodogram is computed, i.e., the discrete Fourier transform of the data contained in that very block. The spectral density is then estimated by taking the average over the periodograms. We assume that the periodograms, as random variables, are independent and identically distributed, and that each periodogram is distributed as the square of a Gaussian variable. Then the Welch's estimate of the spectral density is distributed as a (rescaled) $\chi^2(k)$ variable with M degrees of freedom. Denoting as $f_0(\lambda_j)$ the Welch's estimate for the spectral density and as $f(\lambda_j)$ its true value, then we can obtain a confidence interval by applying a tail bound of a $\chi^2(k)$ variable. For example we can exploit the tail bounds (see e.g. [3])

$$\Pr\left\{ f(\lambda_j) < \frac{f_0(\lambda_j)}{1+t} \right\} \leq e^{-Mt^2/8} \,, \tag{35}$$

$$\Pr\left\{ f(\lambda_j) > \frac{f_0(\lambda_j)}{1-t} \right\} \leq e^{-Mt^2/8} \,. \tag{36}$$

For $t \ll 1$ this yields, up to higher order terms,

$$\Pr\left\{ f(\lambda_j) \notin [(1-t) f_0(\lambda_j), (1+t) f_0(\lambda_j)] \right\} = P(t) \tag{37}$$

with

$$P(t) \leq 2\, e^{-Mt^2/8} \,. \tag{38}$$

Let us first discuss the estimation of the entropy rate

$$h(X) = \frac{1}{2} \int_0^{2\pi} \frac{d\lambda}{2\pi} \log\left[2\pi e f(\lambda)\right] , \tag{39}$$

as approximated by the finite sum

$$h(X) \simeq \frac{1}{2}\sum_{j=1}^{n}\frac{1}{n}\log\left[2\pi e f(\lambda_j)\right]. \tag{40}$$

For each given j, $1-P(t)$ is the probability that $f(\lambda_j) \in [(1-t)f_0(\lambda_j), (1+t)f_0(\lambda_j)]$, then it follows (from an application of the union bound) that

$$\Pr\left\{\exists j \mid f(\lambda_j) \notin [(1-t)f_0(\lambda_j), (1+t)f_0(\lambda_j)]\right\} \leq nP(t). \tag{41}$$

This is equivalent to say that, with probability larger than $1-nP(t)$, $f(\lambda_j)$ lays between $(1-t)f_0(\lambda_j)$ and $(1+t)f_0(\lambda_j)$ for all $j = 1, \ldots, n$. Therefore

$$h(X) \in \left[\frac{1}{2}\sum_{j=1}^{n}\frac{1}{n}\log\left[2\pi e f_0(\lambda_j)\right] + \frac{1}{2}\log(1-t), \frac{1}{2}\sum_{j=1}^{n}\frac{1}{n}\log\left[2\pi e f_0(\lambda_j)\right] + \frac{1}{2}\log(1+t)\right] \tag{42}$$

with probability at least equal to $1-nP(t) = 1-2ne^{-Mt^2/8}$. A further linear approximation for $t \ll 1$ yields the confidence interval

$$h(X) \in \left[\frac{1}{2}\sum_{j=1}^{n}\frac{1}{n}\log\left[2\pi e f_0(\lambda_j)\right] - \frac{\log e}{2}t, \frac{1}{2}\sum_{j=1}^{n}\frac{1}{n}\log\left[2\pi e f_0(\lambda_j)\right] + \frac{\log e}{2}t\right]. \tag{43}$$

Finally, to take into account the overlap between adjacent periodograms, we replace $M \to \gamma M$, for $\gamma < 1$. For example, if the periodogram have a 50% overlap we put $\gamma = 1/2$. In conclusion, with an overlap of 50%, we obtain that for any given $\epsilon > 0$, the entropy rate lies within the interval

$$h(X) \simeq \frac{1}{2}\sum_{j=1}^{n}\frac{1}{n}\log\left[2\pi e f_0(\lambda_j)\right] \pm 2\log e\sqrt{\frac{1}{M}\ln\left(\frac{2n}{\epsilon}\right)}, \tag{44}$$

up to a probability not larger than ϵ.

From the entropy rate we obtain a confidence interval for the conditional variance, $\sigma_X \in [\sigma_X^-, \sigma_X^+]$, where

$$\sigma_X^{\pm} = \frac{1}{2\pi e}2^{\sum_{j=1}^{n}\frac{1}{n}\log\left[2\pi e f_0(\lambda_j)\right]}\,2^{\pm 4\log e\sqrt{\frac{1}{M}\ln\frac{2n}{\epsilon}}}. \tag{45}$$

Similarly, we obtain an estimate of the signal variance σ^2 by exploiting the relation

$$\sigma^2 = \int_0^{2\pi}\frac{d\lambda}{2\pi}f(\lambda), \tag{46}$$

from which we derive a confidence interval

$$\sigma^2 \simeq \left(1 \pm 4\sqrt{\frac{1}{M}\ln\frac{2n}{\epsilon}}\right)\sum_{j=1}^{n}\frac{1}{n}f_0(\lambda_j). \tag{47}$$

Along the same lines we obtain a confidence interval for the conditional noise variance, $\sigma_U \in [\sigma_U^-, \sigma_U^+]$ (this must additionally includes systematic errors). To obtain a worst-case estimate of the min-entropy we consider the smaller value for the signal variance, σ_X^-, and the larger one for the noise, σ_U^+.

V. SUPPLEMENTARY NOTE 5: CHARACTERIZATION OF VACUUM FLUCTUATIONS POWER SPECTRAL DENSITY

Here, we describe the homodyne detector and show by including imperfections that the bound given in the main text is indeed always a lower bound on the vacuum fluctuations. As described in the main text we beat two lasers, the local oscillator with power P_{LO} and an auxiliary signal laser with power P_{sig} which is frequency detuned with respect to the local oscillator by ν. The beams interfere at a beam splitter with splitting ratio $R(\nu) : 1-R(\nu)$, where the frequency dependence ν accounts for a

frequency dependent common mode rejection of the homodyne electronics. We furthermore take into consideration the visibility of the interference $\chi \in (0,1]$ and the quantum efficiencies η_1 and $\eta_2 \in (0,1]$ of the two photo diodes.

After photo detection and current subtraction the beat signal current at time t reads

$$i_{\text{beat}}(t) = 2\chi^2(\eta_1+\eta_2)\sqrt{R(\nu)(1-R(\nu))}\frac{e}{\hbar\omega}\sqrt{P_{\text{LO}}P_{\text{sig}}}\cos(2\pi\nu t)\ . \tag{48}$$

Here ω is the absolute angular frequency of the local oscillator laser. The square of the root mean square (RMS) amplitude of the beat signal digitized by an analog-to-digital (ADC) converter as obtained by a power spectrum of acquired samples is then given by

$$\widetilde{\text{TF}}(\nu) := \left(\sqrt{2}\chi^2(\eta_1+\eta_2)\sqrt{R(\nu)(1-R(\nu))}\frac{e}{\hbar\omega}\right)^2 P_{\text{LO}}P_{\text{sig}}G(\nu)\ , \tag{49}$$

where $G(\nu)$ describes the overall gain of homodyne detector, possible filters and ADC analog input as well as includes the digitization into integers. We call $\text{TF} := \widetilde{\text{TF}}/P_{\text{sig}}$ the transfer function.

The power spectral density (PSD) of the vacuum fluctuations after photo detection and digitization reads

$$\text{PSD}_{\text{vac}} = 2e(i_{\text{dc1}}+i_{\text{dc2}})G(\nu) = 2\frac{e^2}{\hbar\omega}\left(\eta_1(1-R(\nu))+\eta_2R(\nu)\right)P_{\text{LO}}G(\nu)\ , \tag{50}$$

where i_{dc1} and i_{dc2} are the direct photo currents generated by the photo diodes. Using the characterization of the transfer function from Eq. (49) yields

$$\text{PSD}_{\text{vac}} = \hbar\omega\frac{1}{\chi^2}\frac{\eta_1(1-R(\nu))+\eta_2R(\nu)}{(\eta_1+\eta_2)^2R(\nu)(1-R(\nu))}\frac{\widetilde{\text{TF}}(\nu)}{P_{\text{sig}}} \geq \hbar\omega\frac{\widetilde{\text{TF}}(\nu)}{P_{\text{sig}}}\ . \tag{51}$$

In the last step we lower bounded the PSD of the vacuum fluctuations by using $1/\chi \geq 1$ and

$$\left(\eta_1(1-R(\nu))+\eta_2R(\nu)\right)/\left((\eta_1+\eta_2)^2R(\nu)(1-R(\nu))\right) \geq 1\ , \tag{52}$$

where equality holds for $\eta_1 = \eta_2 = 1$, $R = 0.5$.

VI. SUPPLEMENTARY NOTE 6: CONTINUOUS-VARIABLE QUANTUM KEY DISTRIBUTION APPLICATION EXAMPLE

Continuous-variable quantum key distribution uses a Gaussian modulation of the coherent state excitation, i.e. of both the amplitude and phase quadrature components. For a single execution of the protocol a large amount of coherent quantum states, say 10^{10} are generated and transmitted. For each quantum state 2 random numbers are required, the amplitude and the phase quadrature values. In practise these are discretized and for our example we choose an 8 bit resolution. Thus, 16 bit are required to generate 1 coherent state. Since our QRNG delivers 1024 random bits per single execution of the randomness extraction, $N = \frac{16\text{bit}\cdot 10^{10}}{1024\text{bit}} \approx 1.6\times 10^8$ runs are needed.

We now assume that the QRNG ran N_1 times prior to the generation of the random numbers for QKD. Then the random number string for QKD has an epsilon stemming from the hashing of

$$\epsilon_{\text{hash}}^{\text{QKD}} = (N_1+1)\epsilon_{\text{hash}} + (N_1+2)\epsilon_{\text{hash}} + \ldots + (N_1+N)\epsilon_{\text{hash}} = \left(N\cdot N_1 + \frac{(N\cdot(N+1))}{2}\right)\epsilon_{\text{hash}}\ .$$

In our example we require that the random numbers used in the quantum key distribution system have an epsilon security parameter of less than 10^{-9}. Assuming that the parameter estimation epsilon is constant over time and noticing that ϵ_{PE} is one order of magnitude smaller, the series of random numbers must have an epsilon of $\epsilon_{\text{hash}}^{\text{QKD}} \leq 10^{-9}$ from hashing.

After continuously running the QRNG for 10 years,

$$N_1 = \frac{10\,\text{years}}{\frac{352}{1\text{GS/s}}} \approx 9.0\times 10^{14}\ ,$$

where 352 is the number of acquired samples from the ADC per extraction run.

Solving the above equation for ϵ_{hash} and plugging in numbers for N_1 and N yields $\epsilon_{\text{hash}} \lesssim 10^{-32}$.

SUPPLEMENTARY REFERENCES

[1] Weedbrook, C. *et al.* Gaussian quantum information. *Reviews of Modern Physics* **84**, 621–669 (2012).
[2] Seshadreesan, K. P., Lami, L. & Wilde, M. M. Rényi relative entropies of quantum Gaussian states. *Journal of Mathematical Physics* **59**, 072204 (2018).
[3] Lupo, C., Ottaviani, C., Papanastasiou, P. & Pirandola, S. Continuous-variable measurement-device-independent quantum key distribution: Composable security against coherent attacks. *Physical Review A* **97**, 052327 (2018).
